# BRACHYURY and CDX2 Mediate BMP-Induced Differentiation of Human and Mouse Pluripotent Stem Cells into Embryonic and Extraembryonic Lineages

**DOI:** 10.1016/j.stem.2011.06.015

**Published:** 2011-08-05

**Authors:** Andreia S. Bernardo, Tiago Faial, Lucy Gardner, Kathy K. Niakan, Daniel Ortmann, Claire E. Senner, Elizabeth M. Callery, Matthew W. Trotter, Myriam Hemberger, James C. Smith, Lee Bardwell, Ashley Moffett, Roger A. Pedersen

**Affiliations:** 1 The Anne McLaren Laboratory for Regenerative Medicine, University of Cambridge, Cambridge CB2 0SZ, UK; 2 Department of Pathology, University of Cambridge, Cambridge CB2 1QP, UK; 3 Laboratory of Developmental Genetics and Imprinting, The Babraham Institute, Cambridge CB22 3AT, UK; 4 MRC National Institute for Medical Research, Mill Hill, London NW7 1AA, UK; 5 Department of Developmental and Cell Biology, University of California, Irvine, CA 92697-2300, USA; 6 Centre for Trophoblast Research, University of Cambridge, Cambridge CB2 3EG, UK; 7 Department of Zoology, University of Cambridge, Cambridge CB2 3EJ, UK; 8 Department of Physiology, Development and Neuroscience, University of Cambridge, Cambridge CB2 3EG, UK; 9 Department of Surgery, University of Cambridge, Cambridge CB2 0QQ, UK

## Abstract

BMP is thought to induce hESC differentiation toward multiple lineages including mesoderm and trophoblast. The BMP-induced trophoblast phenotype is a long-standing paradox in stem cell biology. Here we readdressed BMP function in hESCs and mouse epiblast-derived cells. We found that BMP4 cooperates with FGF2 (via ERK) to induce mesoderm and to inhibit endoderm differentiation. These conditions induced cells with high levels of BRACHYURY (BRA) that coexpressed CDX2. BRA was necessary for and preceded CDX2 expression; both genes were essential for expression not only of mesodermal genes but also of trophoblast-associated genes. Maximal expression of the latter was seen in the absence of FGF but these cells coexpressed mesodermal genes and moreover they differed in cell surface and epigenetic properties from placental trophoblast. We conclude that BMP induces human and mouse pluripotent stem cells primarily to form mesoderm, rather than trophoblast, acting through BRA and CDX2.

## Graphical abstract

**Graphical Abstract:**

**Figure:**
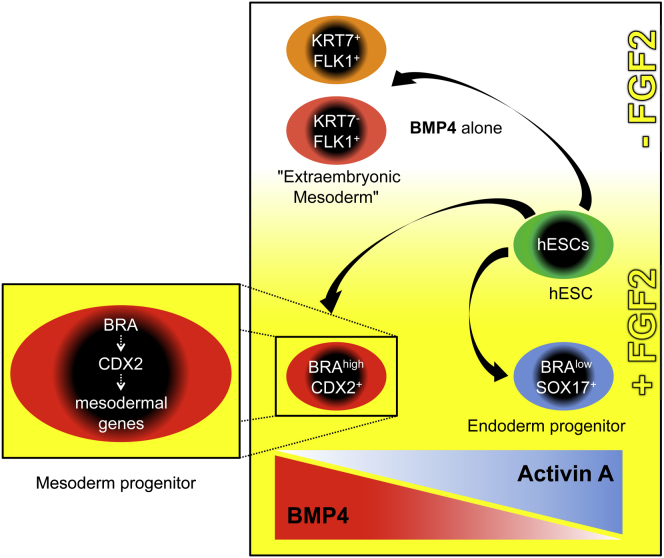

**Highlights:**

► BMP+FGF induces ESCs to express BRA^hi^/CDX2^+^ while Activin+FGF induces BRA^low^/SOX17^+^ ► BRA and CDX2 are required for mesoderm- and trophoblast-associated gene expression ► Epigenetic and surface features discern BMP-treated hESCs from in vivo trophoblast ► BMP-treated hESCs expressing trophoblast-associated genes coexpress mesoderm genes

## Introduction

The mesoderm lineage gives rise to the heart, blood, muscle, kidney, and components of most other somatic tissues, plus placental mesenchyme. Cell-based therapy and disease modeling of any of these derivatives thus relies on a thorough understanding of mesodermal origins. Embryonic and extraembryonic mesoderm, together with definitive endoderm, emerge during gastrulation via an epithelial-mesenchymal transition of epiblast cells at the primitive streak. In the mouse, epiblast cells poised to enter the distal primitive streak are subject to high Nodal signaling and contribute to definitive endoderm. More proximal epiblast cells are subject to high bone morphogenetic protein (BMP) signaling and contribute extensively to mesoderm, including extraembryonic mesoderm, which will form the umbilical cord, the placental mesenchyme, and components of the yolk sack and amnion (Lawson et al., 1991; Parameswaran and Tam, 1995; Kwon et al., 2008; Burtscher and Lickert, 2009).

Human embryonic stem cells (hESCs) represent a promising alternative system for understanding the mechanisms controlling early human development. Studies of hESCs have already provided unequivocal evidence for an endoderm-inducing effect of Activin (D'Amour et al., 2005) and have suggested that BMP signaling is a key inducer of mesoderm (Schneider et al., 2003; Goldman et al., 2009; Zhang et al., 2008; Yang et al., 2008). However, BMP4 also induces the expression of genes associated with trophoblast (Xu et al., 2002) and extraembryonic endoderm (Vallier et al., 2009), and it cooperates with Activin to induce differentiation of mesendoderm, the precursor of mesoderm and endoderm (Vallier et al., 2009). The apparent capacity of hESCs to differentiate into trophoblast and extraembryonic endoderm in response to BMP4 is paradoxical, because they are derived by culturing the inner cell mass (ICM) of expanded blastocysts. At this stage, the inner cells (precursors of epiblast and primitive endoderm) of mouse embryos no longer have the capacity for differentiation into trophectoderm (Tarkowski et al., 2010) and indeed mESCs only rarely colonize the trophectoderm in chimeras (Beddington and Robertson, 1989). Moreover, mouse EpiSCs, which are derived from pre-gastrula-stage late epiblast, also express trophoblast-associated genes in response to BMP (Brons et al., 2007; Tesar et al., 2007; Vallier et al., 2009). These observations raise the question of whether the trophoblast-like phenotype induced by BMP in hESCs and EpiSCs represents an artifact related to their derivation and culture (Rossant, 2008; Silva and Smith, 2008) or alternatively models development of the intact mammalian embryo by inducing expected progeny of the late epiblast. The latter hypothesis would account for the role of BMP4 in the mouse embryo, since homozygous BMP4 mutants have severe defects in embryonic and extraembryonic mesoderm, but none in trophoblast (Winnier et al., 1995).

BMP4, alone or together with Activin, rapidly induces hESCs to express the transcription factor *BRACHYURY* (*BRA*) and other genes characteristic of mesendoderm, the common progenitor of mesoderm and endoderm (Zhang et al., 2008; Yang et al., 2008; Vallier et al., 2009). Progressive differentiation of these cells is evident in expression of genes associated with definitive endoderm, such as *SOX17* and *FOXA2*, or with mid/distal and proximal mesoderm, such as *TBX6* or *FLK1* (Murry and Keller, 2008). Growth factors responsible for mesoderm differentiation of hESCs are not well understood, as BMP does not appear to be the sole driver of mesoderm (Vallier et al., 2009). Fibroblast growth factor (FGF) in particular has been used to promote mesoderm specification and proliferation (Yang et al., 2008; Yook et al., 2011). Understanding which growth factors distinguish between mesoderm, endoderm, and trophoblast differentiation of hESCs is key to their ultimate use for cell-based therapies and disease modeling.

A recent study used FGF2 to modulate the response of hESCs to BMP4, finding that trophoblast-associated genes were induced by BMP only in the absence of FGF (Yu et al., 2011). Although complex trophoblast features, such as giant cell formation and hormone secretion, have been described in BMP-treated hESCs, their trophoblast identity has principally been based on their expression of such trophoblast-associated genes, including *CDX2*, *KRT7*, *HCGα*, and *GCM1* (Xu et al., 2002; Zhang et al., 2008; Vallier et al., 2009; Wu et al., 2008). However, these genes are not exclusively expressed in the trophoblast (Roper and Hemberger, 2009). *CDX2*, for example, not only affects trophoblast development but also plays an essential role in mesoderm (Ralston and Rossant, 2008; Chawengsaksophak et al., 2004; Savory et al., 2009). Indeed, the embryonic expression pattern of *Cdx2* and the postimplantation phenotype of the knockout resemble those of *Bra* (Chawengsaksophak et al., 2004; Savory et al., 2009). Moreover, *Elf5*, a key transcription factor in trophoblast lineage determination (Ng et al., 2008), is not expressed in hESCs, in mEpiSCs or in trophoblast-like cells derived from hESCs (Hemberger et al., 2010). These findings led us to re-examine whether the trophoblast-associated genes induced by BMP are sufficient to identify the resulting phenotype as trophoblast and to exclude epiblast-derived lineages such as embryonic and extraembryonic mesoderm. We studied the response to BMP4 in hESCs, in mEpiSCs, and in explants of mouse late epiblast, using a combination of gene expression analysis, shRNA knockdown, cell surface analysis, and epigenetic characterization. These studies raised doubts that the BMP-induced cells are trophoblast and suggested instead that they are mesodermal in nature.

## Results

### *BRACHYURY* and *CDX2* Expression Are Induced by Cooperative BMP and FGF Signaling

**Figure 1:**
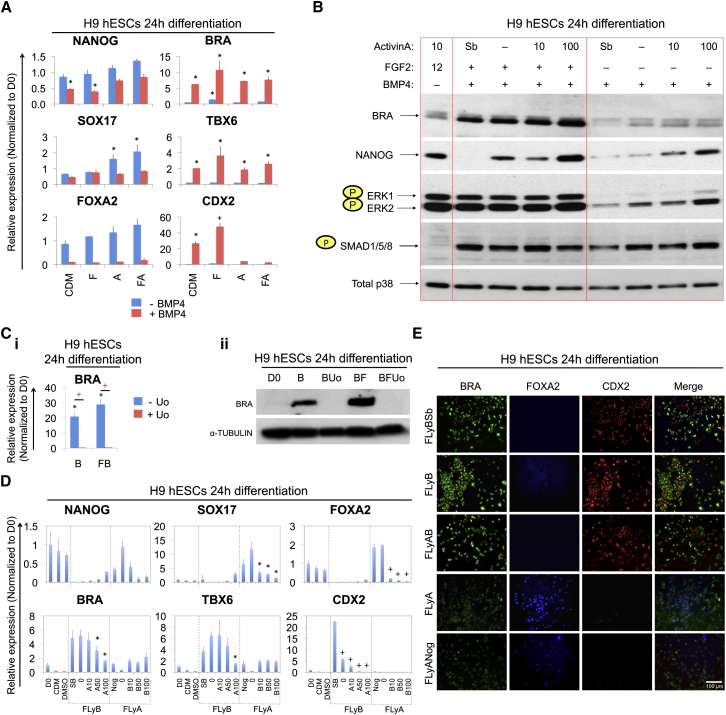
BMP and FGF Cooperate to Induce BRA and CDX2 (A) qPCR analysis of hESCs grown as indicated. Colored bars represent the absence (blue) or presence (red) of BMP4. ^∗^p ≤ 0.05; ^+^p ≤ 0.01; t test. (B) Immunoblots of hESCs grown as indicated, where “Sb” refers to inhibition of Activin/Nodal signaling by SB431542, “-” is no Activin addition and “10” or “100” is the ng/ml Activin. (C) qPCR analysis (i) and immunoblots (ii) of hESCs grown as indicated; “Uo” is ERK inhibitor UO126. In (i) ^∗^p ≤ 0.05; ^+^p ≤ 0.01; t test; comparisons done to day 0 are indicated in black, and comparisons between the two indicated treatments are in red. (D) qPCR analysis of hESCs grown as indicated; pluripotency conditions (day 0, D0), CDM alone, DMSO control, FLyB, or FLyA plus other factors as indicated below each histogram (Nog, Noggin at 200 ng/ml; B, BMP at 10, 50, or 100 ng/ml; Sb, SB431542 at 10 μM; A, Activin at 10, 50, or 100 ng/ml). ^∗^p ≤ 0.05; ^+^p ≤ 0.01; t test. (E) Representative fluorescent images of hESCs grown as indicated. Samples were immunostained for BRA, FOXA2, and CDX2. See also Figure S1.

To understand how growth factor signaling regulates mesoderm differentiation we grew hESCs and mEpiSCs in a chemically defined medium (CDM) that has previously been used to determine the requirements for pluripotency and differentiation (Wiles and Johansson, 1999; Vallier et al., 2005). We used a combinatorial approach to determine the effects of 24 hr treatment with ActivinA (100 ng/ml), BMP4 (10 ng/ml), and/or FGF2 (20 ng/ml), (Figure 1A; Figures S1A and S1B, available online). As expected, Activin treatment of hESCs induced the expression of *SOX17* and *FOXA2*, reflecting endoderm differentiation. By contrast, expression of *BRA*, *TBX6*, and *CDX2* were induced by BMP4, reflecting mesoderm and possibly trophoblast differentiation. This response was most pronounced in the presence of FGF2 and absence of Activin. The critical role of FGF in BMP-induced differentiation was evident when the expression of *BRA* was examined by immunoblotting (Figure 1B). Moreover, omission of FGF or treatment with the FGF receptor inhibitor SU5402 (SU) effectively blocked BMP4-induced *BRA* expression (Figure S1C). These results indicate that FGF, whether provided exogenously or obtained endogenously, was also important for BMP-mediated BRA induction.

To further understand the role of FGF in *BRA* induction we determined which of its effectors mediated this response. We observed high levels of ERK1/ERK2 phosphorylation at 24 hr of FGF treatment (Figure 1B) but did not observe increased phosphorylation of JNK1/JNK2 or p38α (Figure S1D). Furthermore, inhibition of endogenous FGF signaling with SU5402 reduced phospho-ERK below the modest level seen in the absence of added FGF (data not shown). In addition, the ERK inhibitor UO126 (Uo) blocked *BRA* transcript and protein induction by BMP+FGF (Figure 1C). Hence, ERK1/2 activation correlates with FGF signaling and is required for BMP-induced BRA expression. While this study was underway, similar findings were reported on the role of FGF2-ERK in hESCs (Yu et al., 2011).

Independently of FGF, BMP increased the levels of phosphorylated SMAD1/5/8, the transcriptional effectors of canonical BMP signaling (Figure 1B and data not shown). We further found that FGF treatment bypassed the previously described requirement of Activin/Nodal signaling for BMP-induced BRA expression (Nostro et al., 2008) as cells treated with the Activin/Nodal receptor inhibitor SB431542 (Sb) still upregulated BRA protein production, provided that FGF was present (Figure 1B).

The responses to Activin, BMP, and FGF were explored further by simultaneous treatment with LY294002 (Ly), an inhibitor of the PI3 kinase pathway, which facilitates differentiation of hESCs (McLean et al., 2007) (Figure S1E). Induction of endoderm-associated genes (*SOX17* and *FOXA2*) by FGF2 + Ly + Activin (designated “FLyA”) treatment did not require BMP signaling, as demonstrated by use of the BMP inhibitor, Noggin (Figure 1D). Conversely, induction of the mesodermal marker gene *TBX6* and of *CDX2* by FGF2 + Ly + BMP4 (designated “FLyB”) treatment did not require Activin/Nodal signaling, as it was not blocked by Sb (Figure 1D). Immunostaining confirmed these results and highlighted that BMP also induced CDX2 protein expression (Figure 1E). Similar outcomes were observed when mEpiSCs (Figure S1F and data not shown) or outgrowths of late epiblast layers dissected from pre-gastrula stage mouse embryos (Figure S1G) were treated with these conditions. In summary, endoderm-associated gene expression (*SOX17*, *FOXA2*) was induced by Activin + FGF, whereas mesoderm-associated gene expression (*TBX6*) was induced by BMP + FGF in hESCs, in EpiSCs, and in late epiblast outgrowths. The effect of Activin/Nodal, BMP, and FGF on these pluripotent cells thus appears to be consistent with their effect in gastrulating mouse embryos.

### Distinct BRA^high^/CDX2^+^ and BRA^low^/SOX17^+^ Populations Emerge from FLyB and FLyA Treatments

**Figure 2:**
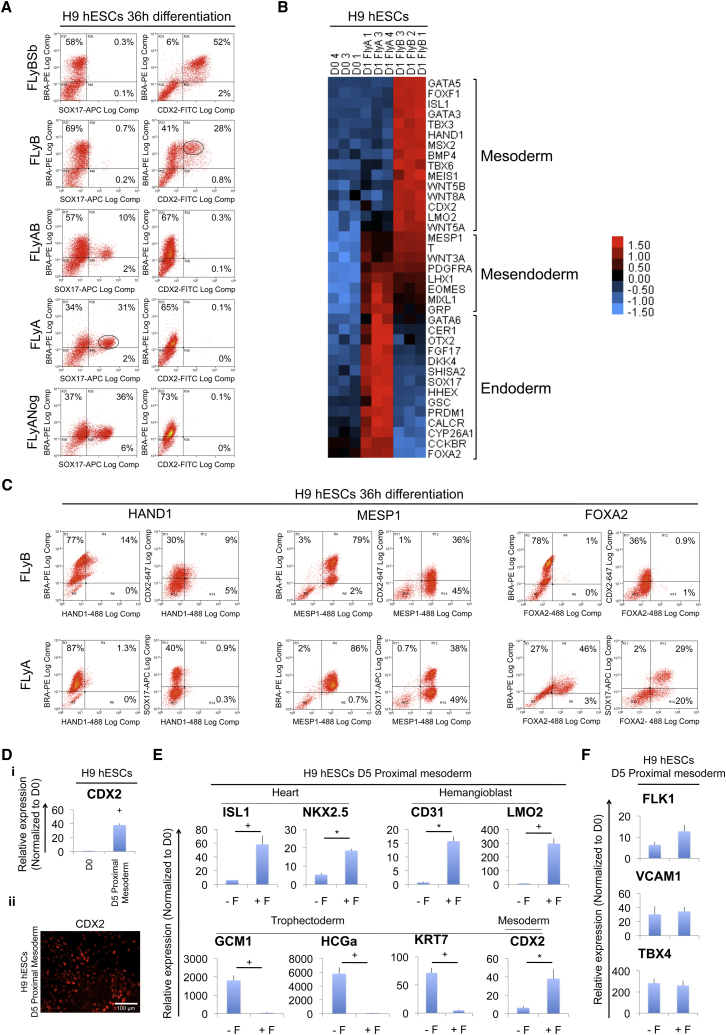
Distinct BRA^high^/CDX2^+^ and BRA^low^/SOX17^+^ Populations Emerge from FLyA and FLyB Treatments (A) Flow cytometry histograms of hESCs grown as indicated. Circles represent subpopulations of BRA^high^ and BRA^low^ cells, which colocalize with CDX2 or SOX17, respectively. (B) Microarray gene expression heat map of undifferentiated (day 0, D0) versus differentiated hESCs grown as indicated. Heat-map colors indicate log_2_ fold-changes. (C) Flow cytometry histograms showing BRA coexpression with HAND1, MESP1, or FOXA2 in hESCs grown as indicated. (Di) qPCR analysis of hESCs induced to differentiate toward proximal streak mesoderm (D5) and untreated controls (pluripotency conditions - D0). (Dii) Representative fluorescent image of D5 hESCs grown as indicated. Samples were immunostained for CDX2. (E) qPCR analysis of hESCs differentiated as indicated. ^∗^p ≤ 0.05; ^+^p ≤ 0.01; t test. Comparisons were done between the two indicated treatments. (F) qPCR analysis of hESCs differentiated as indicated. See also Figure S2.

The reciprocal effects of Activin/Nodal and BMP signaling as inducers of endoderm and mesoderm gene expression, respectively, prompted a deeper examination of the nature of cells in such conditions. As *BRA* mRNA peaked between 24 hr and 36 hr of either FLyA or FLyB treatment (Figure S2A), protein analyses were performed at 36 hr. Flow cytometry analysis showed that FLyA and FLyB treatments each generated 65%–90% BRA expressing cells (Figures 2A and 2C; Figure S2B). However, FLyB induced higher levels of BRA expression than FLyA (Figure 2A; Figure S2C). Moreover, in FLyB-treated hESCs, BRA colocalized with CDX2, while in FLyA-treated hESCs, BRA colocalized with SOX17 (Figure 2A); this result was confirmed in a second hESC line, HuES9 (Figure S2B). Thus, FLyA and FLyB treatments, respectively, evoked two distinct phenotypes: a BRA^low^ population, a majority of which (55% of the total BRA-positive cells) colocalized with SOX17^+^, and a BRA^high^ population, many of which (45% of the total BRA-positive cells) colocalized with CDX2^+^ (Figure 2A).

As levels of Bra expression in the mouse primitive streak have been shown to influence cell recruitment and their phenotype (Wilson and Beddington, 1997), we hypothesized that 36 hr FLyA and FLyB treatments would induce broadly different gene expression profiles. The heat map of predominantly up- or downregulated genes confirmed that characteristic endoderm-associated genes (D'Amour et al., 2005) were upregulated in FLyA and downregulated or absent in FLyB (Figure 2B). Conversely, of numerous genes that were upregulated in FLyB and downregulated or absent in FLyA, many were explicitly mesodermal (e.g., *ISL1*, *TBX6*, *LMO2*) and some were trophoblast associated (e.g., *HAND1*, *CDX2*). Other genes were upregulated in both conditions (e.g., *BRA*, *MIXL1*, *MESP1*, *EOMES)*, suggesting that they distinguish a mesendodermal population. To extend these results, we investigated the protein expression of HAND1, MESP1, and FOXA2 in FLyB and FLyA conditions (Figure 2C; Figures S2D and S2E). Flow cytometry analysis showed that, as suggested by the transcriptional profiles, HAND1 and FOXA2 expression were only observed in BMP or in Activin, respectively. As before, both treatments induced a high proportion of BRA-expressing cells (approximately 80%–90%). HAND1 expression in response to FLyB mainly colocalized with BRA in BRA^high^ cells and also colocalized with CDX2 (65% of HAND1^+^ cells). By contrast, FOXA2 expression in response to FLyA mainly colocalized with BRA in BRA^low^ cells and with SOX17 (65% of FOXA2^+^ cells). As expected from their transcriptional profiles, both FLyB- and FLyA-treated hESCs expressed MESP1, which colocalized with BRA in both BRA^high^ and BRA^low^ cells. Taken together, these results confirmed that mesoderm and endoderm emerged as two distinct populations in response to treatment with BMP + FGF or Activin + FGF, respectively.

The above observations also raised the possibility that trophoblast-associated genes such as CDX2 and HAND1, which are coexpressed with BRA in BRA^high^ cells, are expressed in, and play a role in, mesoderm. To test this hypothesis we subjected 36 hr FLyB-treated cells to a longer mesoderm differentiation protocol by exposing them to an additional 3.5 days of BMP + FGF. *CDX2* expression was induced by this protocol not only in hESCs (Figure 2D) but also in mEpiSCs (Figure S2F) and in epiblast explants (Figure S2G). Since trophoblast gene expression has been reported in hESCs treated with BMP alone (Xu et al., 2002; Yu et al., 2011), we also examined the influence of FGF on expression of *CDX2* and other genes induced by BMP. Omission of FGF2 (Figure 2E) revealed its critical role in promoting the expression of *ISL1*, *NKX2.5*, *CD31*, and *LMO2* in BMP-treated cells. Each of these is integral to development of one or more embryonic mesoderm subtypes. High expression of *CDX2* also depended on FGF signaling and decreased in its absence. In contrast, expression of other trophoblast-associated genes (*GCM1*, *HCGα*, *KRT7*) was highest in the absence of FGF (Figure 2E), as recently also found by Yu et al. (2011). However, *FLK1*, *VCAM1*, and *TBX4*, three BMP-induced genes with essential roles in both embryonic and extraembryonic mesoderm (Yamaguchi et al., 1993; Gurtner et al., 1995; Naiche and Papaioannou, 2003), were also highly expressed in the absence of FGF (Figure 2F). Collectively, the patterns of gene expression in response to BMP either with or without FGF suggest that these two conditions induce distinct molecular phenotypes, but that both are mesodermal in nature.

### Expression of Both Embryonic and Extraembryonic Lineage-Associated Genes in BMP-Treated hESCs Depends on a BRA-Driven Gene Regulatory Network

**Figure 3:**
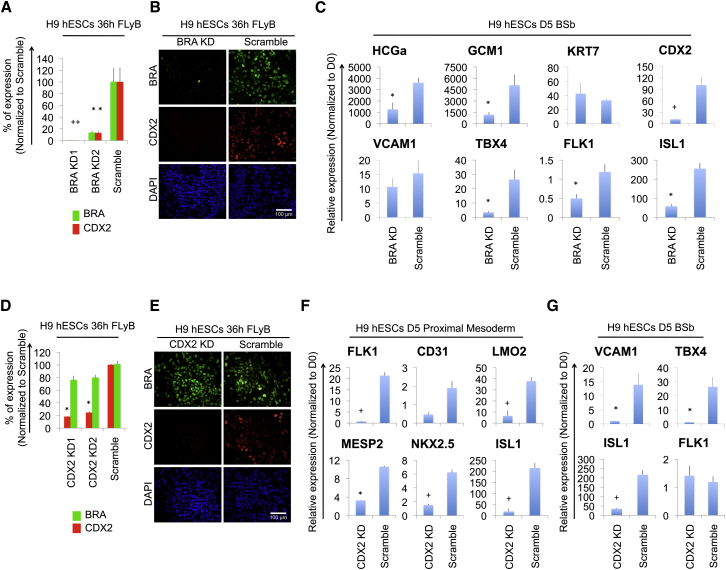
Expression of Both Embryonic and Extraembryonic Lineage-Associated Genes in BMP-Treated hESCs Depends on a BRA-Driven Gene Regulatory Network (A) qPCR analysis of sh-*BRA* knockdown (*BRA* KD) hESC lines and a scrambled control line (Scramble) grown as indicated. ^∗^p ≤ 0.05; ^+^p ≤ 0.01; t test. (B) Representative fluorescent images of *BRA* KD and control hESCs grown as indicated. Samples were immunostained for CDX2, BRA, and DAPI. (C) qPCR analysis of sh-*BRA* KD and control hESCs grown as indicated to induce expression of extraembryonic genes. ^∗^p ≤ 0.05; ^+^p ≤ 0.01; t test. (D) qPCR analysis of sh-*CDX2* knockdown (*CDX2* KD) hESC lines and a control line grown as indicated. ^∗^p ≤ 0.05; ^+^p ≤ 0.01; t test. (E) Representative fluorescent images of *CDX2* KD and control hESCs grown as indicated. Samples were immunostained for CDX2, BRA, and DAPI. (F) qPCR analysis of *CDX2* KD and control hESCs differentiated as indicated. ^∗^p ≤ 0.05; ^+^p ≤ 0.01; t test. (G) qPCR analysis of *CDX2* KD and control hESCs grown as indicated. ^∗^p ≤ 0.05; ^+^p ≤ 0.01; t test. See also Figure S3.

To test the hypothesis that trophectoderm-associated genes are actually expressed in cells of the mesodermal lineage, we determined their dependence on a *BRA*-centered regulatory network. This approach was based on observations that there are no trophoblast phenotypes in homozygous *Bra* mouse mutants (Beddington et al., 1992). We thus evaluated the effect of BMP treatment on hESCs in which *BRA* was stably knocked down using transcript-specific short-hairpin RNAs (sh-*BRA*). As anticipated from its role in mesoderm development in mouse and other species, knockdown of *BRA* in hESCs significantly reduced transcription of key mesoderm genes (*FLK1*, *ISL1*, and *CD31*) (Figure S3A). Interestingly, *BRA* knockdown also nearly completely eliminated *CDX2* expression in FLyB treated cells (Figures 3A and 3B), revealing that *CDX2* induction is dependent on *BRA* in these conditions. Consistent with this, hESCs treated with FLyB expressed *BRA* before they expressed *CDX2*, which accumulated as protein almost exclusively in *BRA*-expressing cells (Figure S3B). Low levels of *CDX2* induction were nevertheless detected in wild-type hESCs treated with Sb or SU (Figure S3C), suggesting that *CDX2* is less dependent on BRA under such conditions. To determine whether *BRA* was required for expression of other BMP-induced extraembryonic genes, the sh-*BRA* knockdown lines were treated for 5 days with BMP + Sb, which induces the highest levels of trophoblast-associated genes (Wu et al., 2008 and Figure S3D) and the lowest levels of mesoderm-associated genes (Figure 1B; Figures S3C and S3E). This revealed that even in BSb conditions the *BRA* knockdown lines were defective in CDX2 as well as in the expression of both trophoblast-associated (*HCGα*, *GCM1*) and mesoderm-associated genes (*FLK1*, *TBX4*, *ISL1*, *LMO2*, and *CD31*) (Figure 3C; Figure S3F).

We then similarly evaluated the role of *CDX2*. Notably, although *BRA* knockdown eliminated *CDX2* expression, *CDX2* knockdown did not reciprocally significantly affect *BRA* expression (Figures 3D and 3E). However, when sh-*CDX2* hESCs were differentiated toward proximal- or mid/distal-streak mesoderm for 5 days, they showed significantly reduced expression of other mesoderm genes, including markers of haemangioblast (*FLK1*, *CD31*, and *LMO2*), heart (*FLK1*, *MESP2*, *NKX2.5*, and *ISL1*), and paraxial mesoderm (*MESP2* and *TBX6*) (Figure 3F; Figure S3G). Interestingly, the *CDX2* knockdown also reduced expression of the trophoblast-associated genes *HCGα*, *GCM1*, and *KRT7* (Figure S3H). Likewise, *CDX2* knockdown lines treated for 5 days with BMP + Sb expressed significantly reduced levels of *TBX4*, *VCAM1*, *ISL1*, *CD31*, and *LMO2* (Figure 3G; Figure S3H). Thus, *CDX2* knockdown affected not only expression of trophoblast-associated genes but also expression of a large panel of mesoderm genes both in the presence and the absence of exogenous FGF signaling.

In summary, the *BRA*-driven gene regulatory network in hESCs includes *CDX2*, and together these are necessary for expression of both mesoderm-associated and trophoblast-associated gene sets.

### Characteristics of BMP-Treated hESCs Distinguish Them from Placental Trophoblast

To resolve the identity of these cells, we examined BMP-treated hESCs further. We focused on well-characterized epigenetic properties of trophoblast cells, namely on the epigenetic status of their *ELF5* promoter (Ng et al., 2008; Hemberger et al., 2010) and on the characteristic repertoire of human leukocyte antigen (HLA) class I molecules expressed by human trophoblast cells in vivo (Apps et al., 2009).

**Figure 4:**
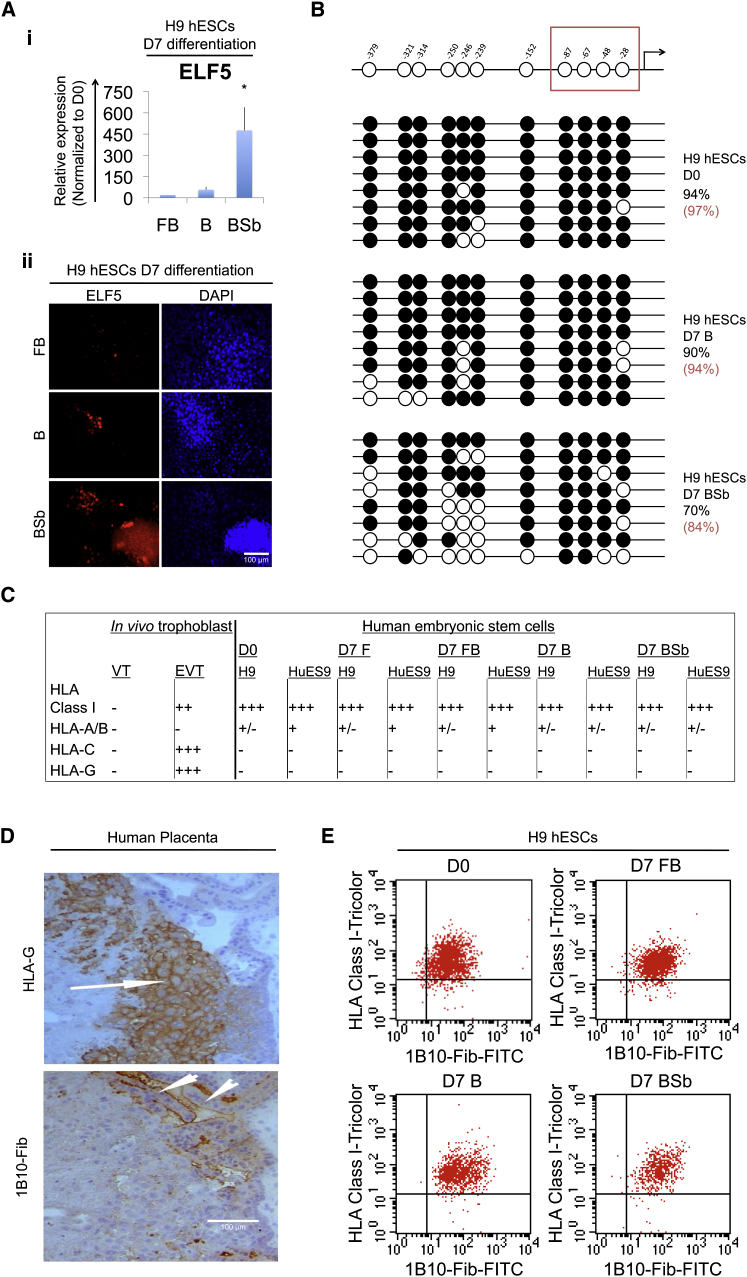
Characteristics of BMP-Treated hESCs Distinguish Them from Placental Trophoblast (Ai) qPCR analysis of ELF5 expression in hESCs grown as indicated. (Aii) Representative fluorescent images of hESCs grown as indicated. Samples were immunostained for ELF5. (B) Bisulphite sequencing analysis of the ELF5 promoter region in hESCs grown as indicated. Filled circles indicate methylated cytosine residues. Top row: hypomethylation status in placental trophoblast. Distance from ATG is shown in base pairs. (C) Table of HLA class I gene expression in placental (in vivo) trophoblast (Apps et al., 2009) and hESCs grown in pluripotency (day 0, D0) or in differentiating conditions as indicated. Legend: -, no expression (0%–4%); +/−, 5%–30%; +, 31%–50%; ++, 51%–70%; +++, 71%–100% of the cells, as determined by flow cytometry. (D) Representative images of placental serial sections stained for HLA-G or the 1B10-fibroblast antigen (1B10-Fib). Arrows indicate HLA-G-positive extravillous trophoblast cells; arrowheads indicate 1B10-fibroblast-negative villous trophoblast. (E) Flow cytometry histograms showing class I HLA expression (detected by W6/32 antibody) and the epitope detected by 1B10-fibroblast antibody (1B10-Fib) in hESCs grown in pluripotency conditions (hESCs) or in differentiating conditions as indicated. See also Figure S4.

In the mouse and human trophoblast lineages, *ELF5* is hypomethylated (≈9%) and highly expressed, but it is hypermethylated and silenced in mEpiSCs and hESCs (Ng et al., 2008; Hemberger et al., 2010). In the absence of FGF, BMP-treated cells expressed some *ELF5*, albeit at low levels (with a cycle threshold [CT] of 32), and ELF5-positive cells comprised only a small fraction of cells (Figure 4A). Consistent with this, the *ELF5* promoter was highly methylated in BMP-treated hESCs and the critical CpG dinucleotides surrounding the transcriptional start site (Hemberger et al., 2010) were hypermethylated in all samples. Slightly lower methylation was seen with BMP + Sb treatment (Figure 4B); this correlated with slightly raised *ELF5* transcript levels, which were still much lower than in placental trophoblast (Hemberger et al., 2010). Nevertheless, the majority of CpGs remained methylated across the *ELF5* promoter.

To further evaluate the cellular identity of BMP-treated hESCs, we assessed the repertoire of HLA class I expression in two independent hESC lines. The two main placental trophoblast subpopulations in vivo are distinguished by their expression of HLA class I molecules. Villous trophoblast does not express any HLA class I molecules while the extravillous population has a unique pattern of expression characterized by the classical HLA-C and nonclassical HLA-E and HLA-G molecules. Highly polymorphic classical HLA-A and HLA-B molecules are not expressed in either trophoblast population (Apps et al., 2009). We found that 75%–95% of BMP-treated hESCs expressed class I HLA molecules, as detected by the W6/32 antibody, which recognizes this entire class. However, while BMP-treated hESCs (with or without FGF or Sb) expressed HLA-A and HLA-B molecules, these cells lacked detectable surface expression of HLA-G or HLA-C (Figure 4C; Figures S4A and S4B). These results suggest that if any trophoblast cells arose from BMP treatment of hESCs, they were a minor population of class I HLA negative cells, which by analogy with in vivo tissues could only be villous trophoblast. We then triple-stained BMP-treated hESCs for class I HLA, for EGFR (a marker of villous cytotrophoblast; Tavaré and Holmes, 1989), and for 1B10-antigen (a marker of villous syncytiotrophoblast; Figure 4D) (Figure 4E). There was no detectable expression of EGFR in the various BMP-treated cultures (data not shown). However, the vast majority (80%–90%) of the BMP-treated hESCs expressed the 1B10-antigen, which is also typically expressed in adult fibroblast tissues of mesodermal origin (Singer et al., 1989). The 1B10 antigen was almost exclusively detected in cells coexpressing HLA class I molecules as detected by W6/32 (Figure 4E). Thus, these data exclude the possibility that an authentic trophoblast population emerges after BMP treatment of hESCs, even in the conditions most favorable to the appearance of such a phenotype.

### Cells Expressing *HCGα*, *GCM1*, and *KRT7* Represent a Subpopulation of Mesoderm Cells

**Figure 5:**
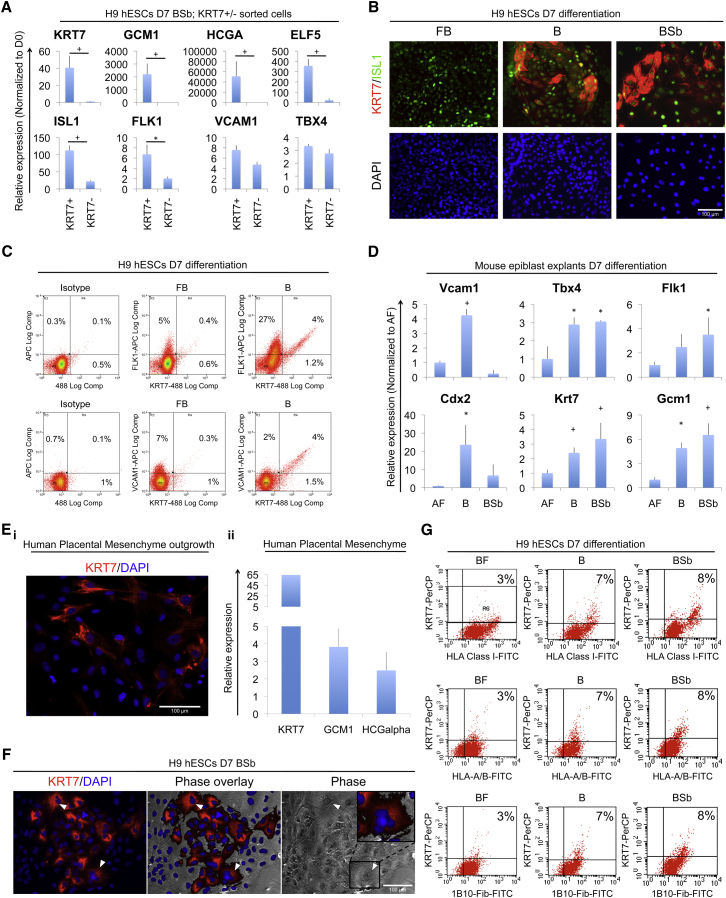
Cells Expressing *HCGα*, *GCM1*, and *KRT7* Represent a Subpopulation of Mesoderm Cells (A) qPCR analysis of KRT7^+^ and KRT7^−^ cells sorted from hESCs grown as indicated. (B) Representative fluorescent images of hESCs grown as indicated. Samples were immunostained for KRT7, ISL1, and the nuclear marker DAPI. (C) Flow cytometry histograms showing FLK1 (upper panels), VCAM-1 (lower panels), and KRT7 coexpression in hESCs grown as indicated. (D) qPCR analysis of mouse late epiblast explants grown in pluripotency (A, ActivinA, 10 ng/ml; F, FGF2, 20 ng/ml) or differentiation conditions as indicated. ^∗^p ≤ 0.05; ^+^p ≤ 0.01; t test. (Ei) Representative fluorescent image of human placental mesenchyme plated for a week in serum-containing medium. Samples were immunostained for KRT7 and with DAPI. (Eii) qPCR analysis of human placental mesenchyme before plating cells to generate outgrowths. (F) Representative light and fluorescent images of hESCs differentiated as indicated. White arrowheads point to multinucleated cells. (G) Flow cytometry histograms showing KRT7 expression, class 1 HLA epitopes (W6/32 antibody), class I A and B HLA epitopes (Tu155 antibody), and the epitope detected by 1B10-fibroblast (1B10-Fib) in hESCs grown as indicated. See also Figure S5.

As BMP-induced cells do not fulfill essential criteria of the trophoblast lineage, it is important to establish their correct lineage identity. All in vivo placental trophoblast subpopulations express *KRT7*, so we used it as a marker for trophoblast-like cells that resulted from BMP treatment of hESCs. Neither H9 nor HuES9 hESC lines expressed KRT7 when grown in CDM supplemented with Activin + FGF, which maintain pluripotency in chemically defined conditions (Figure S5A and S5B). We then sought to determine whether KRT7^+^ cells coexpressed any mesoderm lineage-specific genes when cultured in BMP + Sb, the condition that maximally induced *KRT7*. In each case, there was a small fraction (4%–8%) of BMP-treated cells that expressed KRT7, and virtually all of these coexpressed mesoderm-associated genes. Q-PCR analysis of KRT7^+^ sorted cells revealed expression of *FLK1*, *VCAM1*, and *TBX4* and other mesoderm-associated genes (*CD31* and *LMO2*) (Figure 5A; Figure S5C). KRT7^+^ cells also robustly coexpressed *GCM1* and *HCGα* and expressed *ELF5* at lower levels (Figure 5A). We further verified coexpression of KRT7 protein with ISL1, FLK1, and VCAM1 using immunostaining and flow cytometry and we made similar observations in the HuES-9 hESC line (Figures 5B and 5C; Figures S5D and S5E). In sum, the coexpression of *KRT7* with multiple mesoderm-associated genes indicates that BMP promoted differentiation to a mesodermal phenotype in which a subpopulation of cells expressed genes commonly regarded as markers of the trophoblast lineage, but these cells did not fulfill other criteria of a bona fide trophoblast identity.

We also considered whether the expression of trophoblast-associated genes in response to BMP treatment could be a consequence of derivation and prolonged culture. When explants of mouse late epiblast were treated directly with BMP4, they expressed high levels of Cdx2 and increased expression of *Vcam1*, *Flk1*, and *Tbx4*; they also had increased expression of *Krt7* and *Gcm1* (Figure 5D; Figure S1F). Moreover, explant cultures of human placental mesenchyme that had been purified by cell sorting also expressed *KRT7*, *GCM1*, and *HCGa* (Figure 5E). Thus, in both cases expression of trophoblast-associated genes occurred in the absence of stem cell derivation or prolonged culture.

Finally, we considered whether the multinucleated morphology of sporadic cells in BMP-treated hESCs (Figures S5F and S5G) represented a syncytiotrophoblast-like population abnormally devoid of EGFR expression. Interestingly, such multinucleated cells resembled those appearing after several days (observed from passage 1) in primary cultures of human placental extraembryonic mesenchyme purified by cell sorting (Figure S5H). As syncytiotrophoblast cells are KRT7-positive, 1B10-positive, and HLA class I-negative, we asked whether KRT7 expression colocalized with the syncytiotrophoblast marker 1B10 or with HLA class I molecules (detected by W6/32 antibody) or the specific HLA-A and HLA-B molecules (detected by Tu155 antibody). The multinucleated cells stained positive for KRT7 (Figure 5F). Furthermore, KRT7^+^ cells costained positive for each of these (1B10 and HLA class 1-specific) antibodies (Figure 5G). Despite staining positive for the 1B10 marker, the positive signal for HLA class I molecules thus excludes the possibility that these KRT7-expressing cells are syncytiotrophoblast. Instead, their phenotype suggests a mesodermal identity, possibly corresponding to extraembryonic placental mesenchyme.

## Discussion

Our results reveal how BMP4 and FGF2 (via ERK) cooperate to drive efficient hESC differentiation into early mesodermal cells, which characteristically express high levels of BRA. These cells coexpress CDX2, a gene commonly thought to be trophoblast specific but whose coexpression in cells with BRA suggests instead an either embryonic or extraembryonic mesodermal identity, consistent with patterns of Cdx2 expression observed in the E7.5 mouse embryo (Beck et al., 1995). We further find that Activin/Nodal and FGF2 signaling promotes endoderm differentiation, characterized by lower levels of BRA expression, which often colocalizes with SOX17 and FOXA2 (genes regarded as markers of definitive endoderm and shown here to be induced in early response to FLyA). Mouse EpiSCs and epiblast explants respond similarly to these conditions. Overall, these responses resemble those of the intact mouse embryo, highlighting the likely relevance of the mechanisms underlying in vitro cell fate decisions to in vivo embryonic development and their evolutionary conservation among mammals.

Using sh-RNAs to perturb *BRA* and *CDX2* function, we found that they both regulate mesodermal gene expression, concurring with previous studies in the mouse (Beddington et al., 1992; Chawengsaksophak et al., 2004; Savory et al., 2009). Intriguingly, *BRA* regulates *CDX2* expression in hESCs, but *CDX2* did not regulate *BRA*. Our findings differ from a recent report concluding that *CDX2* regulates *BRA* (Savory et al., 2009), but this may reflect the differing context in which their study was performed (mouse tail bud). Moreover, our results show that both *BRA* and *CDX2* regulate not only genes regarded as mesoderm markers, but also genes previously regarded as trophoblast markers. While *Bra* is regarded as a marker of primitive streak, early mesoderm, node, and notochord, its expression has also been reported in the allantoic core and in extraembryonic ectoderm (Rivera-Pérez and Magnuson, 2005; Inman and Downs, 2006). However, there is no known mouse *Bra* trophoblast phenotype (Beddington et al., 1992). We thus hypothesized either that BMP-induced *BRA* could drive mesodermal expression of genes traditionally associated with the trophoblast lineage or that BMP could induce a subpopulation of bona fide trophoblast, as previously reported (Xu et al., 2002), which relies on *BRA*. Hence, we sought to define culture conditions that would distinguish between these possibilities.

Complementary to the recent report of Yu et al. (2011), we find that BMP-induced hESCs express mesoderm-associated genes (*ISL1*, *NKX2.5*, *CD31*, *LMO2*, and *CDX2*) in the presence of FGF, whereas they express trophoblast-associated genes (*KRT7*, *GCM1*, and *HCGα*) only in the absence of FGF. Importantly, we show that hESC-derived cells induced by BMP to express the trophoblast-associated gene *KRT7* coexpressed mesoderm- and trophoblast-associated genes in single-cell assays (immunostaining and flow cytometry). The BMP4 mutant mouse phenotype adds further support to the hypothesis of a mesodermal identity for BMP-induced hESCs expressing those genes, as it has severe mesoderm defects but no apparent trophoblast phenotype (Winnier et al., 1995). Evidence that FGF8 and FGFR1 mutant mice have defects in embryonic but not extraembryonic mesoderm (Sun et al., 1999; Ciruna and Rossant, 2001) further raises the possibility that BMP-induced, FGF-independent gene expression reflects an extraembryonic mesoderm identity. Indeed, expression of *FLK1*, *VCAM1*, and *TBX4*, which are mesoderm-associated genes with developmental roles in mouse extraembryonic mesoderm, was FGF independent and was observed in KRT7^+^ sorted cells. Moreover, treatments that induced the highest levels of trophoblast-associated genes in hESCs and EpiSCs (BMP alone or BMP + Sb) also induced expression of mesoderm-associated genes. Importantly, these mesoderm genes were not expressed in trophoblast subpopulations, which have been recently isolated and transcriptionally profiled (Apps et al., 2011), thus further showing that the BMP-induced cells do not phenocopy placental trophoblast cells. On the other hand, we readily detected expression of *KRT7*, *GCM1*, and *HCGα* in primary purified human placental mesenchyme cultures (i.e., the extraembryonic mesoderm component of the placenta), indicating that this is not exclusive to placental trophoblast. However, a definitive assessment of the identity of *KRT7*-expressing, BMP-treated hESC-derived cells as either embryonic or extraembryonic mesoderm is difficult, because of the shared expression of numerous mesoderm-associated genes across embryonic and extraembryonic subsets.

The distinction between mesoderm and trophoblast lineages based solely on marker genes is problematic, as many or most of the genes used are dual- or multilineage, rather than lineage-specific, markers. However, we showed that BMP-treated hESCs differed clearly in their pattern of HLA class I expression (and in other cell surface features) from either villous or extravillous placental trophoblast. Furthermore, the high levels of *ELF5* promoter methylation (even in conditions most favorable to trophoblast-associated gene expression) argue against hESC differentiation into cells with a true trophoblast identity (Hemberger et al., 2010). Consistent with this, we observed only low levels of ELF5 expression in BMP-treated hESCs. This is reminiscent of the weak, punctate ELF5 expression in human placenta mesenchymal cells and contrasts with the prominent ELF5 staining in nuclei of villous cytotrophoblast (Hemberger et al., 2010). Taken together, these observations lead us to the conclusion that the BMP-induced phenotype is not bona fide trophoblast but is instead a subpopulation of mesodermal cells.

Lastly, our findings that EpiSCs and mouse epiblast explants respond similarly to hESCs when treated with BMP argues that this response is not a result of tissue culture adaptation, but instead represents an innate property of late (pregastrulation) epiblast and the pluripotent stem cells that share its pluripotent state (Brons et al., 2007; Tesar et al., 2007). Genetically unmodified mESCs can also be induced to express trophoblast-associated genes in some conditions (including BMP4 treatment) (Hayashi et al., 2010; He et al., 2008; Schenke-Layland et al., 2007). As mESCs only rarely contribute to trophoblast tissues (Beddington and Robertson, 1989), their in vitro behavior may indicate that such conditions drive them to another phenotype than trophoblast. In addition, it has not been excluded that those conditions enable mESCs to progress to a late-epiblast-like state, where they would respond similarly to EpiSCs.

In summary, we show here that BMP and FGF cooperate to induce differentiation of hESCs, EpiSCs, and mouse epiblast explants into mesoderm and to inhibit their differentiation into endoderm. Characterization of differentiating cells reveals a role for *CDX2* in human mesoderm development and places *BRA* upstream of *CDX2* and other genes previously regarded as markers of the trophoblast lineage. These findings lead us to question the previous identification of cells induced by BMP treatment of hESCs as bona fide trophoblast. After extensive characterization, we conclude that BMP treatment of hESCs primarily induces a mesodermal phenotype (possibly extraembryonic mesoderm). We cannot exclude the possibility that, as has been shown for mESCs, genetic modification of hESCs might enable the generation of trophoblast from hESCs. Likewise, it is possible that alternative differentiation protocols including the addition of epigenetic modifiers could lead to the generation of trophoblast from hESCs. However, we have no evidence to support the thesis that hESCs have the capacity to generate trophoblast simply by addition of BMP to their growth medium, in either the presence or the absence of FGF2, or with Activin/Nodal inhibition (Xu et al., 2002; Zhang et al., 2008; Vallier et al., 2009; Wu et al., 2008; Yu et al., 2011). Instead, we show that BMP-induced hESCs expressing trophoblast-associated genes share key features with mesoderm. Accordingly, we find that the responses of hESCs and mouse pluripotent stem cells cultured from late epiblast mirror those previously observed in the intact mammalian embryo. In all, this reinforces the utility of pluripotent mammalian stem cells as in vitro models for mammalian development.

## Experimental Procedures

### Human ESC and Mouse EpiSC Culture in Chemically Defined Conditions

Human ESCs (H9 [WiCell, Madison, WI]) and mEpiSCs (129S2-EpiSCs) were grown in a chemically defined medium (CDM) as previously described (Brons et al., 2007). For differentiation, cells were grown in CDM containing PVA instead of BSA and supplemented as described in the figure legends and in the Supplemental Information. Transfection and selection of knockdown lines was done as described in the Supplemental Information.

### Mouse Late Epiblast Layer Dissection and Culture Conditions

Late epiblast layers were dissected from pregastrulation stages (E6.5) of 129S2 mice and cultured as previously described (Brons et al., 2007). All mouse studies complied fully with the UK Animals (Scientific Procedures) Act 1986 as implemented by the University of Cambridge. For differentiation conditions see the Supplemental Information.

### Human Extraembryonic Mesenchyme Isolation and Culture

Placental tissue was obtained from elective terminations of normal pregnancies between 6 and 12 weeks gestation. Ethical approval for the use of these tissues was obtained from the Cambridge Local Research Ethics Committee. Human placental mesenchyme for culture was isolated by negative selection (for details see the Supplemental Information). Recovered mesenchyme was plated down in Hams-F12 (Invitrogen) supplemented with 20% FCS (Invitrogen) and passaged with 0.2% trypsin for up to four passages.

### RNA Extraction, cDNA Synthesis, and Amplification of Mouse Late Epiblast Layer Explant Cultures

Cultured cells were harvested and amplified as described in the Supplemental Information.

### RNA Extraction and Quantitative Polymerase Chain Reaction

Total RNA was extracted using the RNeasy Mini kit (QIAGEN) following manufacturer's instructions. Each sample was treated with RNase-Free DNase (QIAGEN). Half a microgram of RNA was reverse-transcribed using Superscript III reverse transcriptase (Invitrogen). Quantitative polymerase chain reaction (Q-PCR) mixtures were prepared as described (Applied Biosystems, 4385614). Q-PCR reactions were performed in a 7500 Fast ABI instrument following manufacturer's instructions and as described in the Supplemental Information.

### Western-Blot Analysis

Whole-cell protein extraction was done by acetone precipitation of the RLT flow through from the RNeasy columns (QIAGEN) and the extracts' concentrations were measured using a detergent compatible Bradford based kit (BioRad). SDS-PAGE gels (Invitrogen) were run using 10 μg of protein and blotted into nitrocellulose or PVDF membranes using the iBlot (Invitrogen) as described in the Supplemental Information.

### Immunofluorescence

Cells were fixed for 10 min at room temperature in 4% paraformaldehyde (PFA) and immunostained following standard procedures described in the Supplemental Information. Fluorescent images were taken using an Olympus IX71 microscope.

### Flow Cytometry of Intracellular Proteins

Cell suspensions were fixed and stained using the Cytofix-Cytoperm kit (BD Biosciences) and following manufacturer's instructions as described in the Supplemental Information. Cells were analyzed using a Beckman Coulter CyAn_ADP_ flow cytometer and FlowJo software (Becton Dickinson).

### Flow Cytometry of Extracellular Proteins and Flow Sort

Cell suspensions were first incubated with human IgG (Sigma-Aldrich) to block Fcγ receptor-mediated mAb binding and then immunostained as described in the Supplemental Information. Cells were either sorted using the MoFlo MLS high-speed cell sorter (BeckmanCoulter) or analyzed using a FACscan flow cytometer and Cellquest software (Becton Dickinson).

### Bisulphite Sequencing

Cells were lysed in Nuclei Lysis Solution and genomic DNA purification was done using the Wizard Genomic DNA Purification Kit (Promega) and following the manufacturer's instructions. One to two micrograms of genomic DNA was processed for bisulphite conversion using the EpiTect Bisulfite Kit (QIAGEN) and following the manufacturer's instructions. Nested and primary PCR details are in the Supplemental Information. Gel-purified PCR products were cloned using the pGEM-T Easy Vector System (Promega) and sequenced.

### Microarray Analysis

Sample preparation was performed according to manufacturer's instructions (Illumina). Labeled extracts were hybridized to whole-genome bead array assays (HumanWG-6 v3.0 Expression BeadChip) on an Illumina BeadArray reader. Heatmaps of gene expression were created by importing subsets of processed microarray data (for details see the Supplemental Information).

## Accession Numbers

All microarray data are publicly available in MIAME format from the ArrayExpress microarray data repository (European Bioinformatics Institute; E-MTAB-464).

## Supplemental Information

Document S1. Five Figures, Supplemental Experimental Procedures, and Two Tables
