# Supplementary material for: BRACHYURY and CDX2 Mediate BMP-Induced Differentiation of Human and Mouse Pluripotent Stem Cells into Embryonic and Extraembryonic Lineages
Source: Cell Stem Cell. 2011 Aug 5;9(2):144–55. doi: 10.1016/j.stem.2011.06.015 (PMC3567433; doi:10.1016/j.stem.2011.06.015)
Supplement: Document S1. Five Figures, Supplemental Experimental Procedures, and Two Tables [file mmc1.pdf]

Supplemental Information
Cell Stem Cell, Volume *9*

BRACHYURY and CDX2 Mediate BMP-Induced Differentiation of Human and Mouse Pluripotent Stem Cells into Embryonic and Extraembryonic Lineages

Andreia S. Bernardo, Tiago Faial, Lucy Gardner, Kathy K. Niakan, Daniel Ortmann, Claire E. Senner, Elizabeth M. Callery, Matthew W. Trotter, Myriam Hemberger, James C. Smith, Lee Bardwell, Ashley Moffett, and Roger A. Pedersen

Inventory of Supplemental Information:

1. Supplemental Figures
- Figure S1, related to Figure 1
- Figure S2, related to Figure 2
- Figure S3, related to Figure 3
- Figure S4, related to Figure 4
- Figure S5, related to Figure 5

2. Supplemental Experimental Procedures

3. Supplemental References

4. Supplemental tables
- Table S1. Human Primer List
- Table S2. Mouse Primer List

Fig. S1

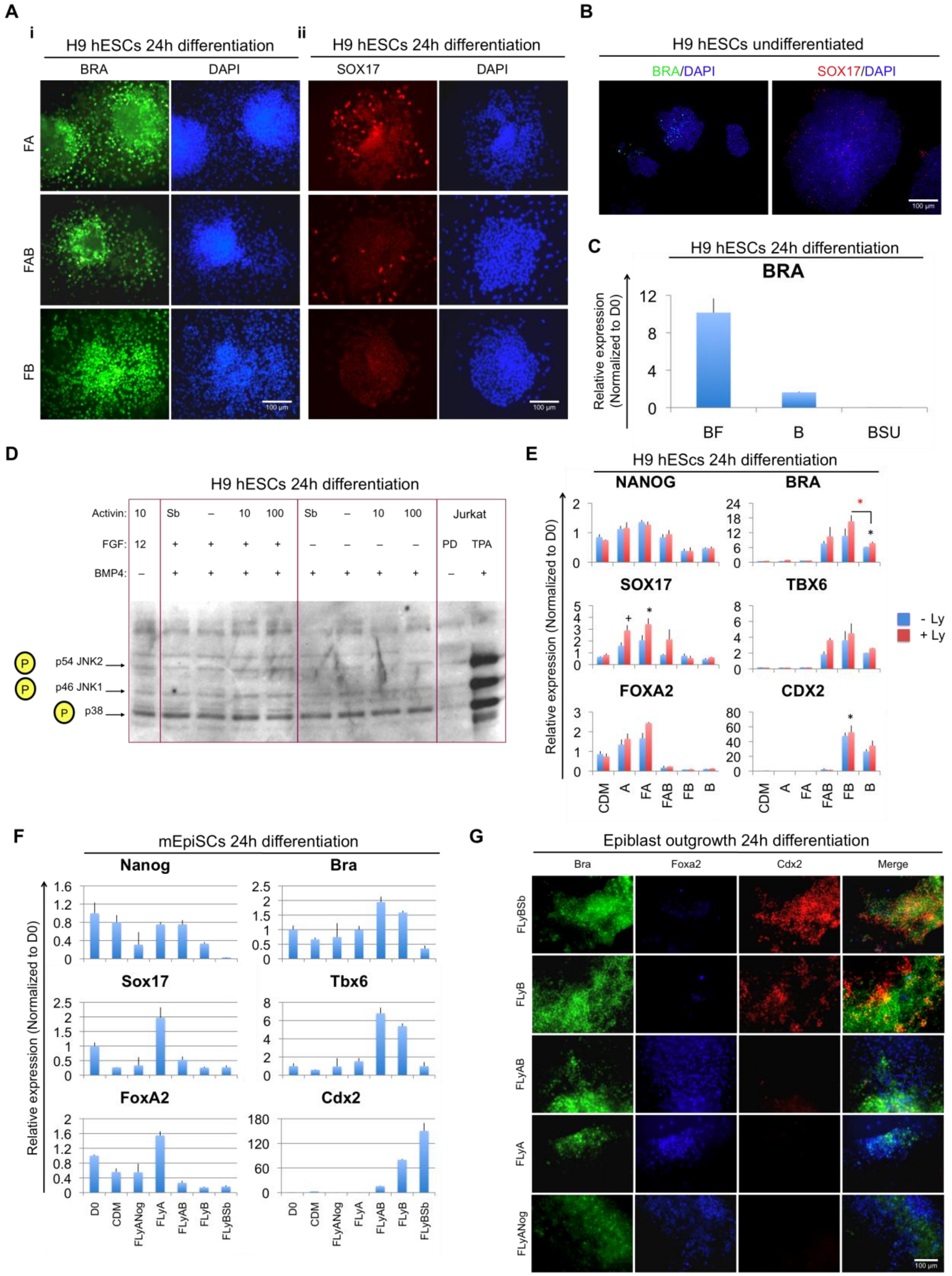
A
i
H9 hESCs 24h differentiation
BRA
DAPI
FA
FAB
FB
100 µm
ii
H9 hESCs 24h differentiation
SOX17
DAPI
100 µm
B
H9 hESCs undifferentiated
BRA/DAPI
SOX17/DAPI
100 µm
C
H9 hESCs 24h differentiation
BRA
Relative expression (Normalized to D0)
12
8
4
0
BF
B
BSU
D
H9 hESCs 24h differentiation
Activin: 10 Sb – 10 100 Sb – 10 100
FGF: 12 + + + + – – – –
BMP4: – + + + + + + + +
Jurkat
PD TPA
P p54 JNK2
P p46 JNK1
P p38
E
H9 hESCs 24h differentiation
NANOG
BRA
SOX17
TBX6
FOXA2
CDX2
Relative expression (Normalized to D0)
- Ly
+ Ly
CDM A FA FAB FB B
F
mEpiSCs 24h differentiation
Nanog
Bra
Sox17
Tbx6
FoxA2
Cdx2
Relative expression (Normalized to D0)
D0 CDM FLyANog FLyA FLyAB FLyB FLyBSb
G
Epiblast outgrowth 24h differentiation
Bra
Foxa2
Cdx2
Merge
FLyBSb
FLyB
FLyAB
FLyA
FLyANog
100 µm

Fig. S1 ***BMP and FGF cooperate to induce BRA and CDX2.*** **A.** Representative fluorescent images of H9 hESCs grown for one day in CDM supplemented as indicated (B, BMP4, 10ng/ml; F. FGF2, 20 ng/ml; A, ActivinA, 100 ng/ml). Samples were immunostained for BRA (i) or SOX17 (ii) and the nuclear marker DAPI. **B.** Representative fluorescent images of undifferentiated H9 hESCs. Samples were immunostained for BRA or SOX17 and the nuclear marker DAPI as indicated. **C.** qPCR analysis of H9 hESCs grown for one day in CDM + BMP4 (B, 10 ng/ml) plus or minus FGF2 (F, 20ng/ml) or the FGF signalling inhibitor SU5402 (SU, 10 µM). **D.** Western blot analysis of pJNK1, pJNK2 and p38 expression in H9 hESCs grown for one day in CDM supplemented with BMP4 (10 ng/ml) plus or minus FGF2 (20 ng/ml) and a gradient of Activin/Nodal signalling (absence, 10 or 100 ng/ml), where 'Sb' refers to SB431542 treatment, '-' refers to no Activin A addition and '10' or '100' refer to the amounts of Activin A in ng/ml). Jurkat cells treated with PD or TPA were used as negative and positive controls, respectively. **E.** qPCR analysis of H9 hESCs grown for one day in CDM alone or supplemented with FGF2 (F, 20ng/ml), Activin A (A, 10ng/ml), BMP4 (B, 10ng/ml), Ly294002 (Ly, 10µM) or combinations of these factors. Coloured bars represent the absence (blue) or presence (red) of Ly294002. T-test was performed where $^{*}p \leq 0.05$; $^{+}p \leq 0.01$; comparisons done to day 0 are indicated in black, and comparisons between the two indicated treatments are indicated in red. **F.** qPCR analysis of undifferentiated control mEpiSCs (D0) or mEpiSCs differentiated for one day in CDM alone or supplemented with FLyA either with (FLyANog) or without Noggin (Nog, 200 ng/ml), FLyAB, FLyB or FLyBSb. T-test was performed where $^{*}p \leq 0.05$; $^{+}p \leq 0.01$. **G.** Representative fluorescent images of dissected mouse late epiblast layers grown for 36h in CDM supplemented as indicated (F, Ly, Sb, Nog as above; B, BMP 10 ng/ml; A, Activin, 100 ng/ml). Samples were immunostained for Bra, Foxa2 and Cdx2. (Related to Fig. 1)

Fig. S2

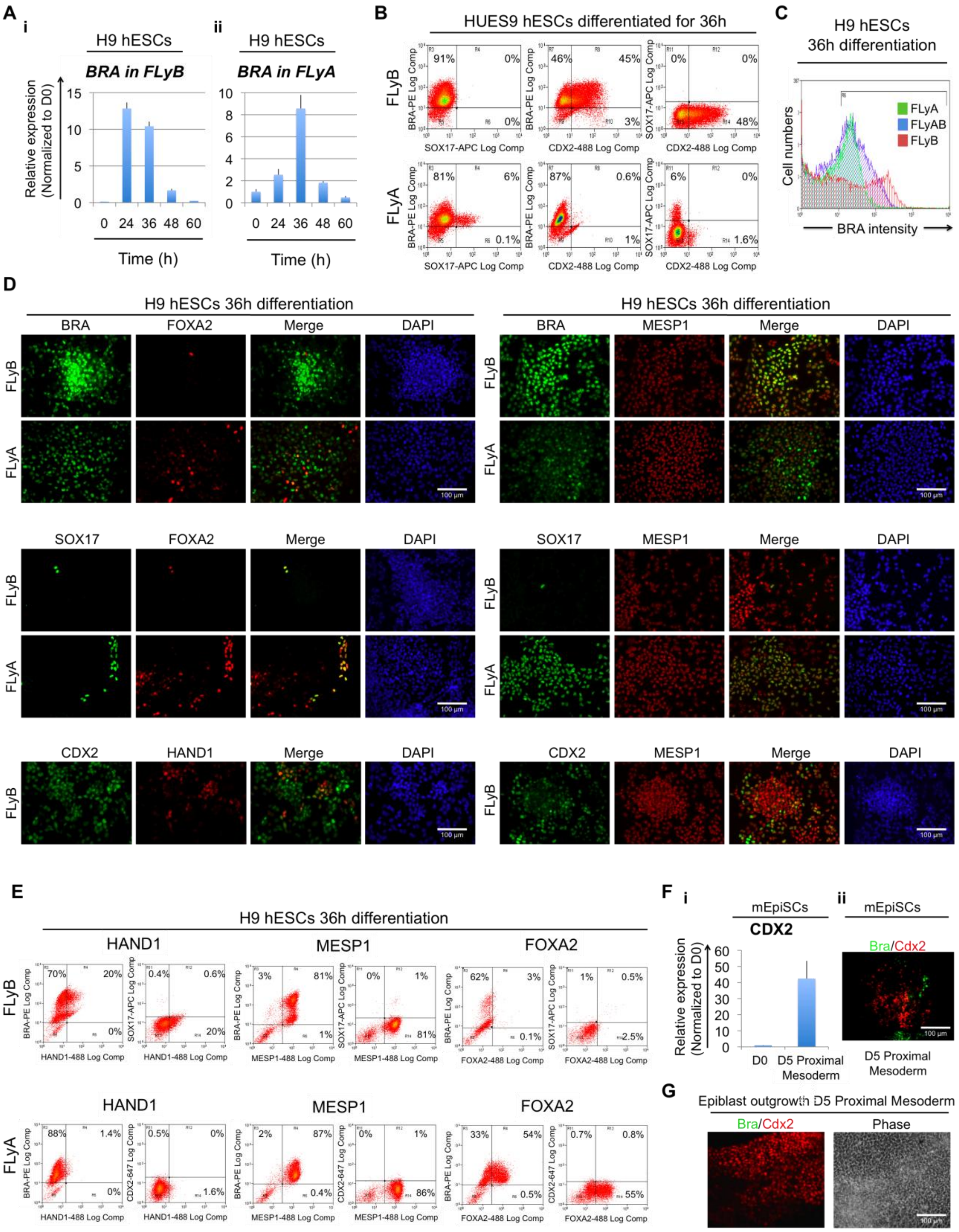
A
i
H9 hESCs
BRA in FLyB
Relative expression (Normalized to D0)
Time (h)
ii
H9 hESCs
BRA in FLyA
B
HUES9 hESCs differentiated for 36h
FLyB
FLyA
SOX17-APC Log Comp
CDX2-488 Log Comp
BRA-PE Log Comp
C
H9 hESCs 36h differentiation
FLyA
FLyAB
FLyB
Cell numbers
BRA intensity
D
H9 hESCs 36h differentiation
BRA
FOXA2
MESP1
Merge
DAPI
SOX17
CDX2
HAND1
100 µm
E
H9 hESCs 36h differentiation
HAND1
MESP1
FOXA2
F
i
mEpiSCs
CDX2
D0
D5 Proximal Mesoderm
ii
Bra/Cdx2
G
Epiblast outgrowth D5 Proximal Mesoderm
Phase

Fig. S2 ***Distinct BRA^{high}/CDX2^{+} and BRA^{low}/SOX17^{+} populations emerge from FLyB and FLyA treatments.*** **A.** qPCR analysis of H9 hESCs differentiated for 0-72h in CDM supplemented with FLyB (i) or FLyA (ii). **B.** Flow cytometry histograms showing BRA expression and its co-expressing genes CDX2 and SOX17 in HUES9 hESCs grown for 36h in CDM supplemented as indicated (FLyB or FLyA, where F was FGF (20 ng/ml), Ly was Ly294002 (10 μM), B was BMP (10 ng/ml) and A was Activin (100ng/ml). **C.** Histogram showing the levels of BRA expression as measured by flow cytometry in H9 hESCs differentiated for 36h in FLyB, FLyAB or FLyA. Note higher levels of BRA in some cells with FLyB treatment and intermediate levels in FLyAB, as compared to FLyA treatment. **D.** Representative fluorescent images of H9 hESCs grown for 36h in CDM supplemented as indicated (F, Ly, Sb as above; B, BMP 10 ng/ml; A, Activin, 100 ng/ml). Samples were immunostained for BRA, FOXA2, CDX2, SOX17 and/or HAND1. **E.** Flow cytometry histograms showing BRA expression and its co-expressing genes HAND1, MESP1 and FOXA2 in H9 hESCs grown for 36h in CDM supplemented with FLyB or FLyA. **Fi.** qPCR analysis of mEpiSCs differentiated for 5 days towards proximal streak mesoderm (see Experimental Methods). **Fii.** Representative fluorescence images of mEpiSCs differentiated for 5 days (D5) towards proximal streak mesoderm, as in Fi. Samples were immunostained for Bra and Cdx2. **G.** Representative fluorescent images of explanted mouse late epiblast layers grown for 5 days in conditions as Fi. Samples were immunostained for Bra and Cdx2. (Related to Fig. 2)

Fig. S3

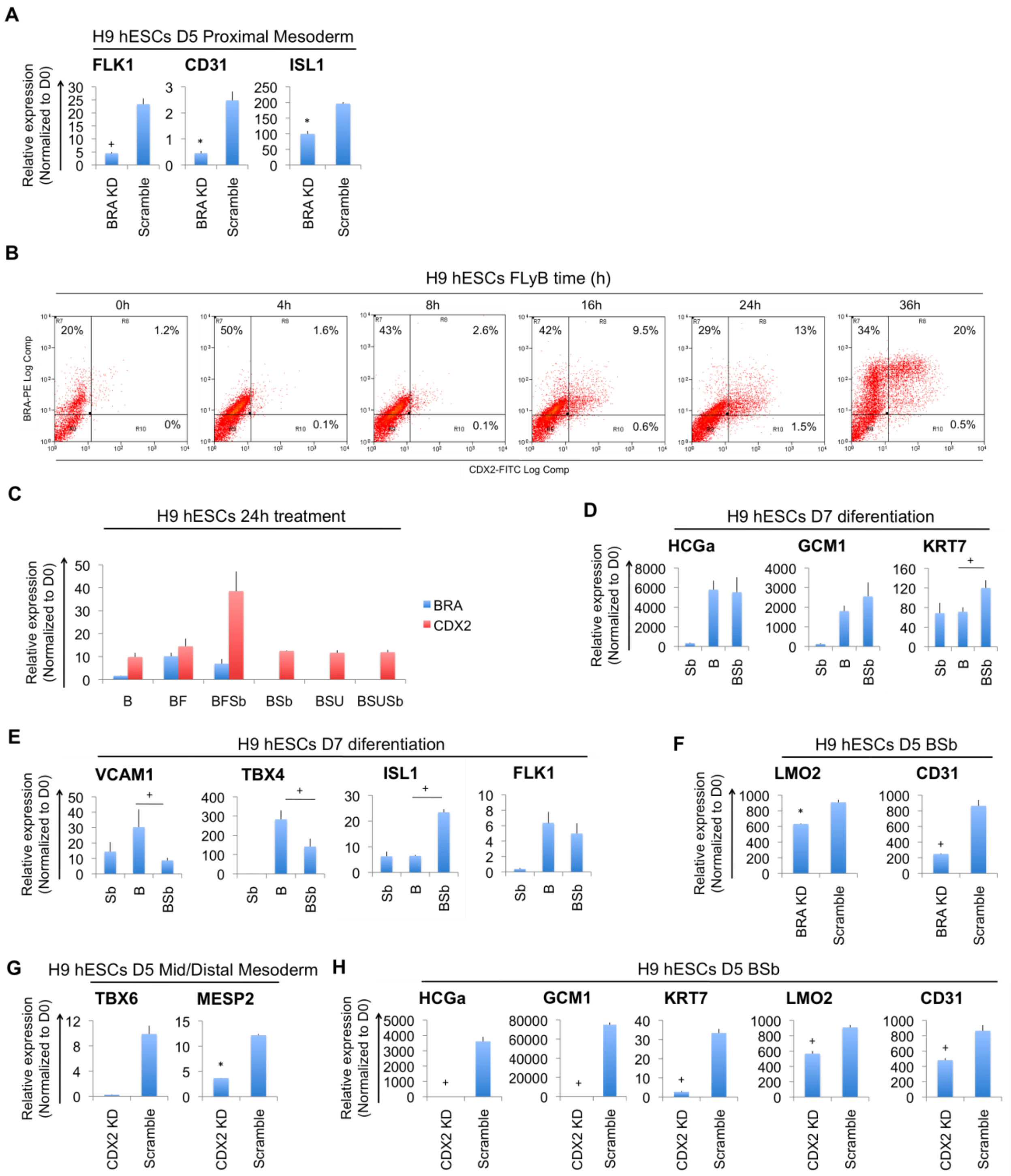

A
H9 hESCs D5 Proximal Mesoderm
FLK1
CD31
ISL1
Relative expression (Normalized to D0)
BRA KD
Scramble
B
H9 hESCs FLyB time (h)
0h
4h
8h
16h
24h
36h
20%
1.2%
0%
50%
1.6%
0.1%
43%
2.6%
0.1%
42%
9.5%
0.6%
29%
13%
1.5%
34%
20%
0.5%
BRA-PE Log Comp
CDX2-FITC Log Comp
C
H9 hESCs 24h treatment
BRA
CDX2
B
BF
BFSb
BSb
BSU
BSUSb
D
H9 hESCs D7 diferentiation
HCGa
GCM1
KRT7
Sb
B
BSb
E
H9 hESCs D7 diferentiation
VCAM1
TBX4
ISL1
FLK1
F
H9 hESCs D5 BSb
LMO2
CD31
G
H9 hESCs D5 Mid/Distal Mesoderm
TBX6
MESP2
CDX2 KD
Scramble
H
H9 hESCs D5 BSb
HCGa
GCM1
KRT7
LMO2
CD31

Fig. S3 ***Expression of both embryonic and extraembryonic lineage-associated genes in BMP-treated hESCs depends on a BRA-driven gene regulatory network.*** **A.** qPCR analysis of *BRA* KD and control (Scramble) H9 hESCs differentiated towards proximal-streak mesoderm (see Experimental Procedures). T-test was performed where $*p \leq 0.05$; $^{+}p \leq 0.01$. **B.** Flow cytometry histograms showing BRA expression and its co-expressing genes SOX17 and CDX2 in H9 hESCs grown for 0-36h in CDM supplemented as indicated (FLyB, where F was FGF (20 ng/ml), Ly was Ly294002 (10 µM) and B was BMP (10 ng/ml) and for the length of time indicated. **C.** qPCR analysis of H9 hESCs grown for 24h in CDM supplemented as indicated where B was BMP (10 ng/ml); F was FGF (20 ng/ml); Sb was SB431542 (10 µM); and SU was SU5402 (SU, 10 µM). T-test was performed where $*p \leq 0.05$; $^{+}p \leq 0.01$. **D.** qPCR analysis of H9 hESCs grown for 7 days in CDM+Sb, CDM+B or CDM+BSb. Expression of trophoblast-associated genes was determined. T-test was performed where $*p \leq 0.05$; $^{+}p \leq 0.01$. **E.** qPCR analysis of H9 hESCs grown for 7 days in CDM+Sb, CDM+B or CDM+BSb, where B was BMP4 (10 ng/ml) and Sb was SB431542 (10 µM). Expression of mesoderm-associated genes was determined T-test was performed where $*p \leq 0.05$; $^{+}p \leq 0.01$. **F.** qPCR analysis of *BRA* KD and control (Scramble) H9 hESCs differentiated in CDM+BSb. T-test was performed where $*p \leq 0.05$; $^{+}p \leq 0.01$. **G.** qPCR analysis of *CDX2* KD and control (Scramble) H9 hESCs differentiated towards distal-streak mesoderm (see Experimental Procedures). **H.** qPCR analysis of sh-*CDX2* KD and control H9 hESCs grown as in F. T-test was performed where $*p \leq 0.05$; $^{+}p \leq 0.01$**.** (Related to Fig. 3)

Fig. S4

A

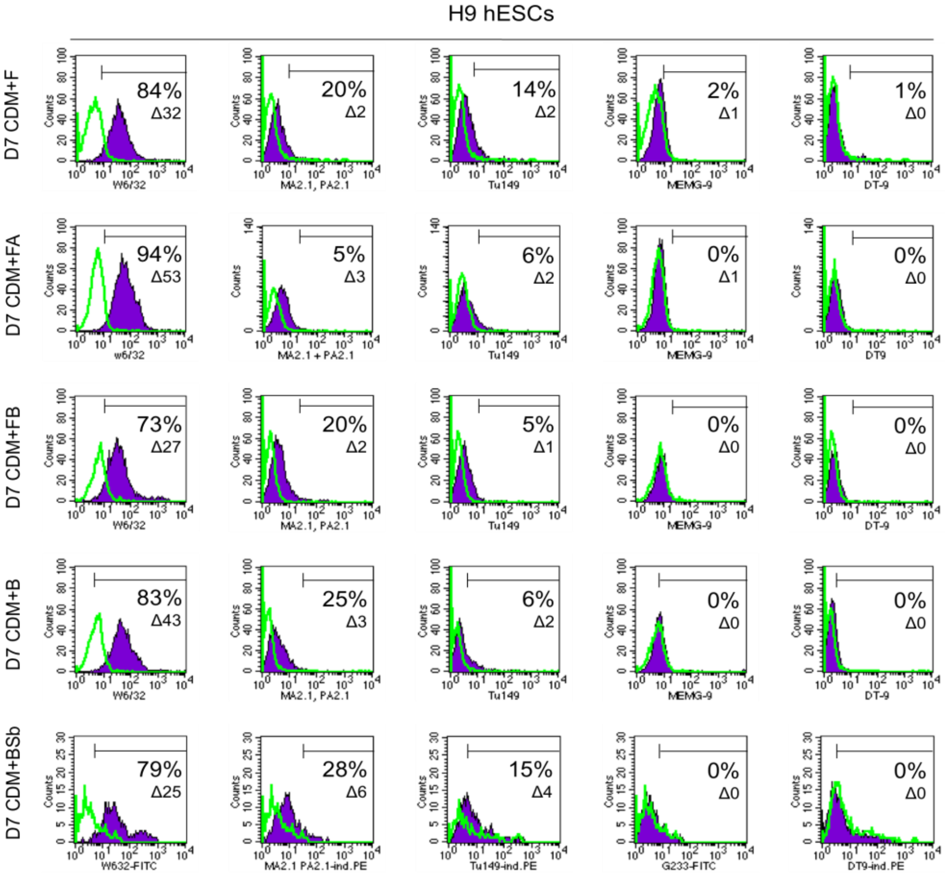
H9 hESCs
D7 CDM+F
84%
Δ32
20%
Δ2
14%
Δ2
2%
Δ1
1%
Δ0
D7 CDM+FA
94%
Δ53
5%
Δ3
6%
Δ2
0%
Δ1
0%
Δ0
D7 CDM+FB
73%
Δ27
20%
Δ2
5%
Δ1
0%
Δ0
0%
Δ0
D7 CDM+B
83%
Δ43
25%
Δ3
6%
Δ2
0%
Δ0
0%
Δ0
D7 CDM+BSb
79%
Δ25
28%
Δ6
15%
Δ4
0%
Δ0
0%
Δ0
Counts

B

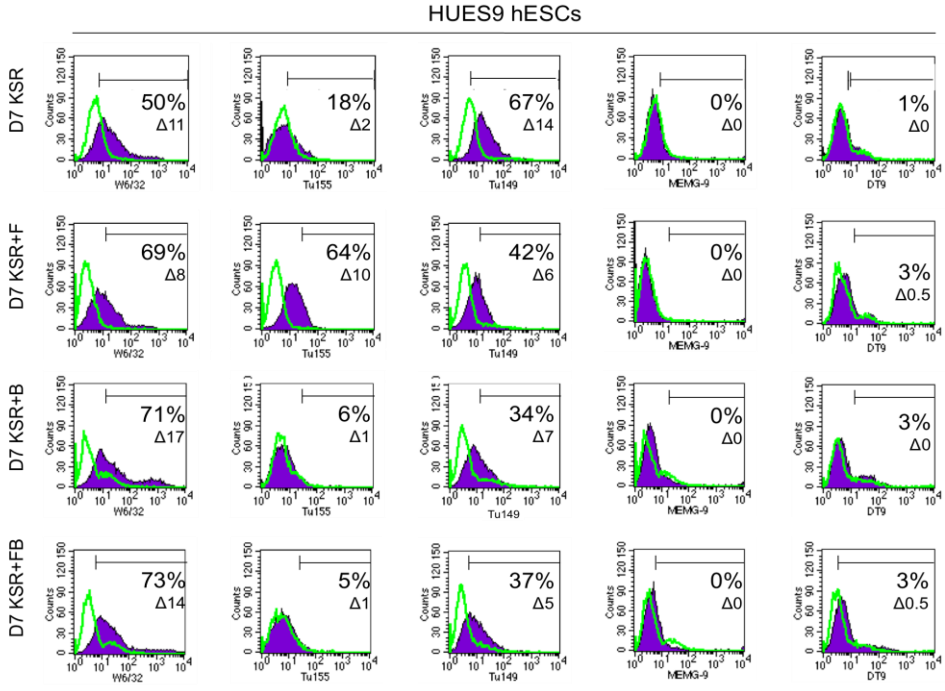
HUES9 hESCs
D7 KSR
50%
Δ11
18%
Δ2
67%
Δ14
0%
Δ0
1%
Δ0
D7 KSR+F
69%
Δ8
64%
Δ10
42%
Δ6
0%
Δ0
3%
Δ0.5
D7 KSR+B
71%
Δ17
6%
Δ1
34%
Δ7
0%
Δ0
3%
Δ0
D7 KSR+FB
73%
Δ14
5%
Δ1
37%
Δ5
0%
Δ0
3%
Δ0.5
Counts

Fig. S4 ***Characteristics of BMP-treated hESCs distinguish them from placental trophoblast.*** **A.** Flow cytometry histograms showing the levels (X-axis) and number of cells (Y-axis) displaying HLA Class I antigens indicated in H9 hESCs grown for 7 days in pluripotency conditions (CDM+F+A, where F is FGF2, 12 ng/ml, and A is Activin, 10 ng/ml) or in CDM supplemented with FGF2 (F, 20ng/ml), BMP4 (B, 10ng/ml), SB431542 (Sb, 10 μM) or combinations of these. The delta fluorescence intensity between the isotype control (green) and the stained samples (purple) is presented, as is the percentage of expressing cells. HLA-A was detected by MA2.1/PA2.1 antibody; HLA-B by Tu149 antibody; HLA-G by by MEMG-9 and G233 antibodies and HLA-C by DT9 antibody. **B.** Flow cytometry histograms showing the levels (X-axis) and number of cells (Y-axis) displaying HLA Class I antigens indicated in HUES9 hESCs grown for 7 days in KSR alone or supplemented with FGF2 (F, 20ng/ml) (pluripotency conditions), in BMP4 (B, 10ng/ml) or both FGF2+BMP4 (FB). The delta fluorescence intensity between the isotype control (green) and the stained samples (purple) is presented, as is the percentage of expressing cells. HLA-A was detected by Tu155 antibody; HLA-B by Tu149 antibody; HLA-G by by MEMG-9 antibody and HLA-C by DT9 antibody. (Related to Fig. 4)

Fig. S5

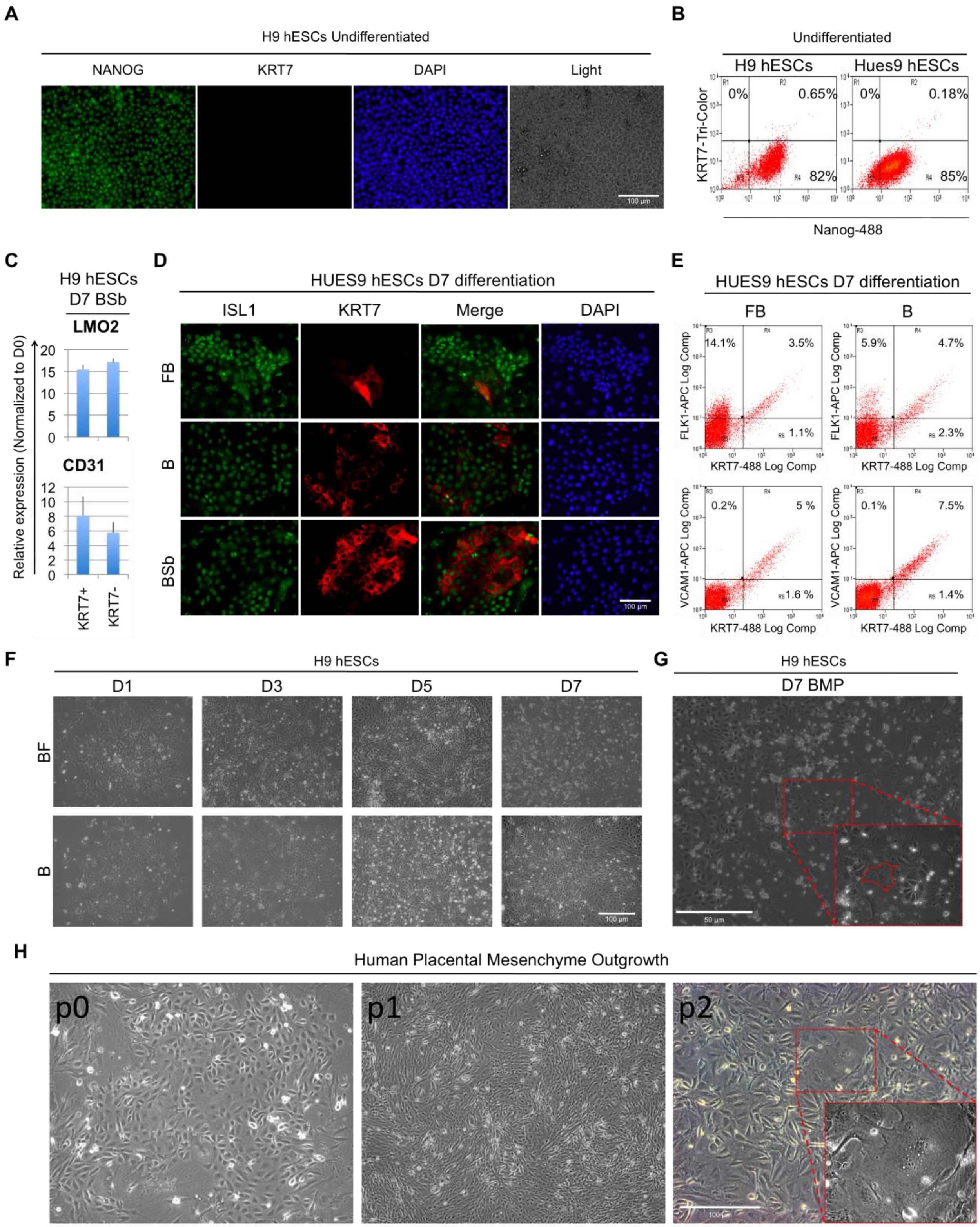

A
H9 hESCs Undifferentiated
NANOG
KRT7
DAPI
Light
100 µm
B
Undifferentiated
H9 hESCs
Hues9 hESCs
KRT7-Tri-Color
0%
0.65%
82%
0%
0.18%
85%
Nanog-488
C
H9 hESCs D7 BSb
LMO2
CD31
Relative expression (Normalized to D0)
KRT7+
KRT7-
D
HUES9 hESCs D7 differentiation
ISL1
KRT7
Merge
DAPI
FB
B
BSb
100 µm
E
HUES9 hESCs D7 differentiation
FB
B
FLK1-APC Log Comp
14.1%
3.5%
1.1%
5.9%
4.7%
2.3%
KRT7-488 Log Comp
VCAM1-APC Log Comp
0.2%
5 %
1.6 %
0.1%
7.5%
1.4%
F
H9 hESCs
D1
D3
D5
D7
BF
B
100 µm
G
H9 hESCs
D7 BMP
50 µm
H
Human Placental Mesenchyme Outgrowth
p0
p1
p2
100 µm

Fig. S5 ***Cells expressing HCGα, GCM1 and KRT7^{+} represent a subpopulation of mesoderm cells.*** **A.** Representative fluorescent and light images of H9 hESCs grown in pluripotency conditions. Samples were immunostained for NANOG, KRT7 and the nuclear marker DAPI. **B.** Flow cytometry histograms showing NANOG and KRT7 expression in H9 and HUES9 hESCs grown in pluripotency conditions. **C.** qPCR analysis of KRT7^{+} and KRT7^{-} cells sorted from H9 hESCs grown for 7 days in CDM supplemented with BMP (10ng/ml) and Sb (10 µM). **D.** Representative fluorescent images of HUES9 hESCs grown for 7 days in CDM supplemented as indicated (F, FGF2 at 20 ng/ml; B, BMP at 10 ng/ml; Sb, SB431542 at 10 µM). Samples were immunostained for KRT7, ISL1 and the nuclear marker DAPI. **E.** Flow cytometry histograms showing FLK1, VCAM and KRT7 expression in HUES9 hESCs grown for 7 days in CDM supplemented as in D. **F.** Representative light images of H9 hESCs differentiating in CDM supplemented as indicated (F, FGF2 at 20 ng/ml; B, BMP at 10 ng/ml; Sb, SB431542 at 10 µM). **G.** Representative high magnification image of a multinucleated cell in H9 hESCs differentiating in CDM supplemented with BMP at 10 ng/ml. **H.** Representative light images of placental extra-embryonic mesenchyme outgrowths expanded for up to 2 passages (p). (Related to Fig. 5)

Supplemental Experimental Procedures

Human ESC and Mouse EpiSC Culture in Chemically Defined Conditions

For differentiation, H9 hESCs were grown with or without FGF2 (20 ng/ml, zebrafish, recombinant, gift of Marko Hyvönen), LY294002 10 μM (Sigma), Noggin 200 ng/ml (R&D Systems), Dorsomorphin 5 μM (Calbiochem), SB431542 10 μM (Tocris) and/or varying concentrations of BMP4 (R&D Systems) and ActivinA (gift of Dr. Marko Hyvönen) for up to 7 days. Early mesoderm differentiation was induced by FGF2 + LY294002 + BMP4 (10 ng/ml), named FLyB; early endoderm differentiation was induced by FGF2 + LY294002 + ActivinA (100 ng/ml), named FLyA. Proximal streak mesoderm differentiation was induced by treating cells with FLyB for 36h followed by FGF2 + BMP4 (50ng/ml) for 3.5 days, while mid/distal-streak mesoderm differentiation was induced by treating cells with FLyB for 36 hours followed by FGF2 + Ly (1 μM) for 3.5 days containing conditions. Trophoblast-like differentiation was induced by treating cells with BMP (10ng/ml) + Sb for 5 or 7 days. The hESC line HUES9 was grown as previously described (Cowan et al., 2004) or differentiated in CDM-PVA as for H9 hESCs. All experiments were repeated at least twice on different passages of cells to ensure that the patterns of gene expression described were reproducible.

Human ESC knockdown lines

Stable knockdowns of *BRA* and *CDX2* were carried out with pLKO.1-shRNA vector (Sigma) by Lipofectamine transfection following manufacturer's instructions. A Scrambled pLKO.1-shRNA vector (Sigma) was used as a control. Stable clones were screened by PCR and immunostaining and the percentage of knockdown was

determined by comparison to expression in the scrambled control transfected lines.

Mouse Late Epiblast Layers Dissection and Culture Conditions

Late epiblast layers were dissected manually from E6.5 129S2 mouse embryos as previously described (Brons et al., 2007). For differentiation, epiblast layers were grown overnight (up to 24 h) in CDM + Activin + FGF and then moved to CDM supplemented with combinations of the growth factors / inhibitors described above for up to 7 days. All differentiation experiments were repeated at least twice using late epiblast layers from different litters.

Human placental mesenchyme extraction and culture

Human placental mesenchyme for culture was isolated by negative selection performed by immunomagnetic sorting using the following combination of FITC-conjugated antibodies and EasySep FITC isolation kit (Stem Cell Technologies): HLA-G clone MEMG-9-FITC (Serotec) for extravillous trophoblasts, EGFR clone ICR10-FITC (Serotec) for villous trophoblasts, CD31 clone WM59-FITC (Biolegend) for endothelial cells, HLA-DR clone G46-6-FITC (Becton Dickinson) and CD45-FITC (Becton Dickinson) for macrophages and all leukocytes. Fewer than 0.3% trophoblast contaminants remained post sorting.

RNA* extraction, *cDNA synthesis and amplification of mouse late epiblast layer explant cultures

Amplified cDNA was purified using a PCR-purification kit (Qiagen) and following manufacturer's instructions. Ethanol precipitation of the DNA was then performed

to further purify the cDNA samples.

RNA extraction and Quantitative Polymerase Chain Reaction

Each sample was run in duplicate and normalised to Porphobilinogen Deaminase (PBGD) in the same run. Primer sequences can be found in Tables S1 and S2. Error bars on all QPCR graphs represent standard deviation from three independent biological replicates. Student's t tests (two-tailed assuming nonequal variance) were performed; superscripts indicate that all three replicates showed significantly different gene expression ($^{*}p \leq 0.05$; $^{+}p \leq 0.01$) compared to the day zero undifferentiated sample group, except where indicated.

Immunobblot analysis

Membranes were incubated overnight with primary antibody at a concentration of 1:1000 or 1:5000 (pERK1/2, p38, pAKT, pJUN1/2 and pJUNK were from Cell Signalling; NANOG and α-TUBULIN were from Abcam, BRA was from R&D Systems). Secondary antibody incubations (1:10000, HRP-conjugated rabbit anti-goat, goat anti-mouse or goat anti-rabbit from Sigma) were performed at room temperature for 1h30min. Lastly, Dura-West substrate (Thermo Scientific), prepared according to the manufacturer’s instructions, was used to develop the immunobblots prior to X-ray film exposure.

Immunofluorescence

Primary antibody incubations were performed over-night at 4°C as follows: goat anti-Brachyury (1:150, R&D Systems), goat anti-Sox17 (1:250, R&D Systems), mouse anti-Cdx2 (1:150, Biogenex), rabbit anti- Foxa2 (1:100, Cell Signaling), rabbit anti-

Nkx2.5 (1:100, Santa Cruz), mouse anti-Mesp2 (1:50, beta-test antibody R&D Systems), rabbit anti-Isl1 (1:50, Abcam) and mouse anti-KR7 (1:400, Santa Cruz). Fluorescently labelled secondary antibodies (IgG, 1:300) were added for 1 h at room temperature as follows: Alexa 488 donkey anti-goat, Alexa 594 donkey anti-goat, Alexa 350 donkey anti-goat, Alexa 488 donkey anti-mouse, Alexa 594 donkey anti-mouse, Alexa 488 donkey anti-rabbit, Alexa 594 donkey anti-rabbit and Alexa 350 donkey anti-rabbit (Invitrogen). In some instances, DAPI was added to the first wash (Sigma 1:10000).

Flow Cytometry of Intracellular Proteins

Antibodies for intracellular detection were added directly conjugated to a fluorofore: Brachyury-PE (R&D Systems), SOX17-APC (R&D Systems), FLK1-APC (BD-Biosciences), VCAM1-APC (BD-Biosciences) or in subsequent stages for those antibodies which were not directly conjugated (mouse anti-CDX2 at 1:200; mouse anti-KRT7 at 1:300; rabbit anti-FOXA2 at 1:200; goat anti-HAND1 at 1:200; rabbit anti-MESP1 at 1:200 + Alexa 488 donkey anti-mouse, donkey anti-goat or donkey anti-rabbit at 1:400). Where required, an intermediate blocking step was performed. Each antibody incubation was done at 4°C for 30 min in blocking buffer (made in Cytoperm buffer) and was followed by 3 washes in Cytoperm buffer. Lastly, cells were re-suspended in PBS supplemented with 1% BSA. The appropriate isotype controls, primary-antibody omission controls and cells only controls, as well as single positive samples for each channel were included as controls.

Flow Cytometry of Extracellular Proteins

Unlabelled primary antibodies were added and detected with fluorochrome-conjugated anti-mouse IgG or IgM (Sigma-Aldrich). Free secondary antibody-binding sites were blocked with murine immunoglobulin, before staining with directly conjugated mAbs. The panel of antibodies used is as follows: W6/32-FITC (Serotec) to monomorphic determinants on all HLA-Class 1 molecules; MA2.1/and PA2.1 to HLA-A2, HLA-A28, HLA-B57, HLA-B58 only; Tu155 (Ziegler) to almost all HLA-A and HLA-B allotypes (also detects some HLA-C allotypes); Tu149 (Ziegler) to all HLA-B allotypes (also detects some HLA-C allotypes); DT9 (this hybridoma was a kind gift from Veronique Braud) to all HLA-C allotypes, MEMG-9-FITC (Serotec) to HLA-G, leukocyte lineage cocktail (CD3, CD14, CD16, CD19, CD20, CD56 Beckton Dickinson), fibroblast surface marker clone 1B10 (Sigma), BC-1 (gift of Doug Antzak) to an unknown determinant on extravillous trophoblast only at the implantation site; Tra1-81 (Chemicon, MAB4381) to a determinant of pluripotency.

Bisulphite sequencing

Nested PCR was carried out on 10% of the eluted DNA to amplify the –432/–3 bp region of the ELF5-2b promoter. Primary PCR was performed using the following primer pair: forward 5’-GGAAATGATGGATATTGAATTTGA-3’ and reverse 5’-CAATAAAAATAAAAACACCTATAACC-3’. For nested PCR, the primers used were: forward 5’-GAGGTTTTAATATTGGGTTTATAATG-3’ and reverse 5’-ATAAATAACACCTACAAACAAATCC-3’).

Microarray Analysis

Bead level data from all hybridizations was background corrected, using default parameters of the *RMA* algorithm (Irizarry et al., 2003), and summarized using the *beadarray* package for the *Bioconductor* suite of software for the *R* statistical programming environment. Processed sample expression profiles were quantile normalized using the *limma* package for *Bioconductor* prior to analysis of differential regulation between sample groups with the moderated t-statistic of the same package (Wettenhall and Smyth, 2004). In order to reduce errors associated with multiple hypothesis testing on such a scale, the significance *p-values* obtained were converted to corrected *q-values* using the false discovery rate (FDR) method of (Storey & Tibshirani, 2003), as implemented in the *qvalue* package for *Bioconductor*. Differential regulation between two sample groups was deemed significant at a threshold of $q < 0.01$ (FDR 1%). Heatmaps of gene expression were created by importing subsets of processed microarray data (for details see Supplementary Information) into the *Java TreeView* package (Saldanha, 2004).

Supplemental References

Brons, I.G., Smithers, L.E., Trotter, M.W., Rugg-Gunn, P., Sun, B., Chuva de Sousa Lopes, S.M., Howlett, S.K., Clarkson, A., Ahrlund-Richter, L., Pedersen, R.A., et al. (2007). Derivation of pluripotent epiblast stem cells from mammalian embryos. Nature 448, 191-195.

Cowan, C.A., Klimanskaya, I., McMahon, J., Atienza, J., Witmyer, J., Zucker, J.P., Wang, S., Morton, C.C., McMahon, A.P., Powers, D., et al. (2004). Derivation of embryonic stem-cell lines from human blastocysts. N Engl J Med 350, 1353-1356. Epub 2004 Mar 1353.

Irizarry, R.A., Hobbs, B., Collin, F., Beazer-Barclay, Y.D., Antonellis, K.J., Scherf, U., and Speed, T.P. (2003). Exploration, normalization, and summaries of high density oligonucleotide array probe level data. Biostatistics 4, 249-264.

Kurimoto, K., Yabuta, Y., Ohinata, Y., and Saitou, M. (2007). Global single-cell cDNA amplification to provide a template for representative high-density oligonucleotide microarray analysis. Nat Protoc 2, 739-752.

Saldanha, A.J. (2004). Java Treeview--extensible visualization of microarray data. Bioinformatics 20, 3246-3248. Epub 2004 Jun 3244.

Storey, J.D., and Tibshirani, R. (2003). Statistical significance for genomewide studies. Proc Natl Acad Sci U S A 100, 9440-9445. Epub 2003 Jul 9425.

Wettenhall, J.M., and Smyth, G.K. (2004). limmaGUI: a graphical user interface for linear modeling of microarray data. Bioinformatics 20, 3705-3706. Epub 2004 Aug 3705.

Supplemental Tables

Table S1. Human Primer List

Gene	Forward	Reverse
PBGD	ATTACCCCGGGAGACTGAAC	GGCTGTTGCTTGGACTTCTC
NANOG	CATGAGTGTGGATCCAGCTTG	CCTGAATAAGCAGATCCATGG
BRA	TGCTTCCCTGAGACCCAGTT	GATCACTTCTTTCCTTTGCATCAAG
MEOX1	TCTGAGCGCCAGGTCAAAG	CTGAACTTGGAGAGGCTGTGG
TBX6	AGCCTGTGTCTTTCCATCGT	GCTGCCCGAACTAGGTGTAT
FLK1	CCCCAGAAATAAAATGGTATAAAAATG	TTTCACTCACTTCCATAATCGTCA
MESP2	GCAGTGTACCAGGGTCTCTCT	ACTGTGGCTCCAGCACCT
SOX17	AGATGCTGGGCAAGTCGT	GCTTCAGCCGCTTCACC
KRT7	AAGAACCAGCGTGCCAAGT	TCCAGCTCCTCCTGCTTG
CDX2	GGAACCTGTGCGAGTGGAT	TCGATATTTGTCTTTCGTCCTG
FOXA2	ATTGCTGGTCGTTTGTTGTG	CCTCGGGCTCTGCATAGTAG
HCGa	GTGCAGGATTGCCCAGAAT	CTGAGGTGACGTTCTTTTGGA
ISL1	AGATTATATCAGGTTGTACGGGATCA	ACACAGCGGAAACACTCGAT
P-CADH	GACAATGCTCCCATGTTTGA	CGCCCATGATAAGGTAGGTG
CD31	GAGTCCTGCTGACCCTTCTG	ATTTTGCACCGTCCAGTCC
LMO2	CTACTACAAACTGGGCCGGAAG	ACCCGCATTGTCATCTCATAG
GCM1	TGCTGTCTGCTTCTCCGTAA	CACCTATTCGACTCCCCTCA
SOX7	ACGCCGAGCTCAGCAAGAT	TCCACGTACGGCCTCTTCTG
NKX2.5	CAAGTGTGCGTCTGCCTTT	CAGCTCTTTCTTTTCGGCTCTA
ELF5	AGAACATCCGCACACAAGGT	TGCCACTTGTTTTCAAGCAG
TBX4	ATGAGAACAATGCTTTCGGCT	TCACGGGGTATTCTTTGCTC
VCAM1	ATGAAATGGATTCTGTGCCC	TTCTTGCAGCTTTGTGGATG

Table S2. Mouse Primer List

Gene	Forward	Reverse
Pbgd	CCTGGCATACAGTTTGAAATCAT	TTTTTCCAGGGCGTTTTCT
Nanog	CCTGAGCTATAAGCAGGTTAAGACC	TGGATGCTGGGATACTCCAC
Bra	AAGAACGGCAGGAGGATGT	TCACGAAGTCCAGCAAGAAA
Tbx6	ACCGCTACCCTGATTTGGATA	AGATGGGAGAAGGGGCAAAG
Flk1	GGCTCTGGTGGAGGTGGGCT	AACGCCACAGGGAACTCGCC
Sox17	GCTAAGCAAGATGCTAGGCAAGT	TCATGCGCTTCACCTGCT
Krt7	GCCGCAGCAACGCCTCACTA	TCCCCGTGACCCAATCATGCCT
Cdx2	TAGGAAGCCAAGTGAAAACCA	CCTCCTGATGGTGATGTATCG
FoxA2	CCCTTCTCTATCAACAACCTCATGT	GGGTAGTGCATGACCTGTTCGT
Gcm1	GCTTCATCTTTTTCCAGTCCA	CTTTCTTCATGGCTCTTCTTG
Tbx4	TCAGACGCCCCTTACCCGGC	GGAGCAGAGCCAGTCCAGCG
Vcam1	TGGAGGTCTACTCATTCCCTGA	TAGTCTCCCCCTTCAGTAATTCAA
